# A Conserved Role for Human Nup98 in Altering Chromatin Structure and Promoting Epigenetic Transcriptional Memory

**DOI:** 10.1371/journal.pbio.1001524

**Published:** 2013-03-26

**Authors:** William H. Light, Jonathan Freaney, Varun Sood, Abbey Thompson, Agustina D'Urso, Curt M. Horvath, Jason H. Brickner

**Affiliations:** Department of Molecular Biosciences, Northwestern University, Evanston, Illinois, United States of America; National Cancer Institute, United States of America

## Abstract

The interaction of nuclear pore proteins (Nups) with active genes can promote their transcription. In yeast, some inducible genes interact with the nuclear pore complex both when active and for several generations after being repressed, a phenomenon called epigenetic transcriptional memory. This interaction promotes future reactivation and requires Nup100, a homologue of human Nup98. A similar phenomenon occurs in human cells; for at least four generations after treatment with interferon gamma (IFN-γ), many IFN-γ-inducible genes are induced more rapidly and more strongly than in cells that have not previously been exposed to IFN-γ. In both yeast and human cells, the recently expressed promoters of genes with memory exhibit persistent dimethylation of histone H3 lysine 4 (H3K4me2) and physically interact with Nups and a poised form of RNA polymerase II. However, in human cells, unlike yeast, these interactions occur in the nucleoplasm. In human cells transiently depleted of Nup98 or yeast cells lacking Nup100, transcriptional memory is lost; RNA polymerase II does not remain associated with promoters, H3K4me2 is lost, and the rate of transcriptional reactivation is reduced. These results suggest that Nup100/Nup98 binding to recently expressed promoters plays a conserved role in promoting epigenetic transcriptional memory.

## Abstract 2

In yeast and humans, interaction of a nuclear pore protein with promoters alters chromatin structure and allows RNA polymerase II to bind, poising them for faster reactivation for several generations.

## Author Summary

Cells respond to changes in nutrients or signaling molecules by altering the expression of genes. The rate at which genes are turned on is not uniform; some genes are induced rapidly and others are induced slowly. In brewer's yeast, previous experience can enhance the rate at which genes are turned on again, a phenomenon called “transcriptional memory.” After repression, such genes physically interact with the nuclear pore complex, leading to altered chromatin structure and binding of a poised RNA polymerase II. Human genes that are induced by interferon gamma show a similar behavior. In both cases, the phenomenon persists through several cell divisions, suggesting that it is epigenetically inherited. Here, we find that yeast and human cells utilize a similar molecular mechanism to prime genes for reactivation. In both species, the nuclear pore protein Nup100/Nup98 binds to the promoters of genes that exhibit transcriptional memory. This leads to an altered chromatin state in the promoter and binding of RNA polymerase II, poising genes for future expression. We conclude that both unicellular and multicellular organisms use nuclear pore proteins in a novel way to alter transcription based on previous experiences.

## Introduction

The nuclear pore complex (NPC) is a conserved macromolecular structure that mediates the essential transport of molecules between the nucleus and the cytoplasm [1]. The NPC is an 8-fold symmetric channel derived from ∼30 proteins associated with cytoplasmic filaments and a nucleoplasmic “basket” [2],[3]. Natively unstructured NPC proteins rich in phenylalanine-glycine repeats line the channel of the NPC and interactions of these proteins with transport factors facilitates selective transport of proteins and mRNPs [2],[4]–[6]. Proteins that make up the basket-like structure on the nucleoplasmic face of the NPC and the fibrils on the cytoplasmic face of the NPC play key roles in regulating nuclear transport and mRNP remodeling [4],[7].

Nuclear pore proteins also physically interact with chromatin to regulate transcription of certain genes. In *Saccharomyces cerevisiae* and *Drosophila melanogaster*, many active genes physically interact with nuclear pore proteins [8]–[11]. Interaction with the NPC has been proposed to promote stronger transcription [9],[12]–[16], to mediate epigenetic regulation [17]–[19], to promote chromatin boundary activity [20],[21], and to provide negative feedback in signaling pathways [22]. However, the exact biochemical nature of these roles, their generality, and their conservation is unclear.

In yeast, some of the inducible genes that relocate from the nucleoplasm to the NPC upon activation [such as *GAL1* (GenBank Accession CAA84962.1) and *INO1* (GenBank Accession CAA89448.1)] remain at the nuclear periphery for multiple generations after repression, a phenomenon called epigenetic transcriptional memory [17]. The persistent association of genes with the NPC is not associated with transcription, but promotes faster reactivation [17],[18],[23]. In the case of the *GAL* genes, this leads to significantly faster reactivation compared with activation [17],[24]. This is not always true; in the case of the *INO1* gene, perhaps because of the rate at which cells sense the activating signal (inositol starvation) during reactivation, the rate of reactivation is slower than the rate of activation [17],[18]. However, interaction with the NPC after repression specifically promotes *INO1* reactivation because when it is lost, the rate of reactivation is slowed [18].

Active *INO1* and recently repressed *INO1* interact with the NPC by distinct mechanisms. Interaction of active *INO1* with the NPC involves *cis*-acting “DNA zip codes” called gene recruitment sequences (GRSs) in the promoter [15]. Interaction of recently repressed *INO1* with the NPC is independent of the GRSs and requires a different zip code called a memory recruitment sequence (MRS), as well as the histone variant H2A.Z (GenBank Accession CAA99011.1) and the nuclear pore protein Nup100 (GenBank Accession CAA81905.1) [18], which is homologous to Nup98 in metazoa. Whereas GRS-mediated interaction of active *INO1* with the NPC promotes stronger transcription [15], MRS-mediated interaction of recently repressed *INO1* with the NPC promotes incorporation of H2A.Z into the promoter and allows RNA polymerase II (RNAPII) to bind, poising the gene for future reactivation [18]. Mutations in the MRS, loss of H2A.Z, or loss of Nup100 specifically block interaction of recently repressed *INO1* with the NPC, leading to loss of RNAPII from the recently repressed promoter and slower reactivation [18]. However, these mutations have no effect on the rate of initial activation or the ultimate steady-state levels of *INO1* mRNA [17],[18],[23]. Thus, the interaction of genes with the NPC can both promote stronger expression and, by a distinct mechanism, poise recently repressed genes for future reactivation.

Stress-inducible genes utilize a related type of transcriptional memory. Previous exposure of yeast cells to high salt leads to faster activation of many genes induced by oxidative stress [19]. Similar to *INO1* transcriptional memory, this effect persists for four to five generations, suggesting that salt stress establishes an epigenetic change that promotes the rate of activation of these genes. The faster rate of activation of these genes is dependent on the NPC protein Nup42 (GenBank Accession EEU07798.1) [19]. MEME analysis of the promoters of 77 genes exhibiting stress-induced transcriptional memory identified a DNA element very similar to the *INO1* MRS element [19]. *GAL* gene transcriptional memory has been suggested to depend on the NPC-associated protein Mlp1 (GenBank Accession CAA82174.1) [23],[25]. Therefore, although there are some gene-specific features, aspects of the molecular mechanism of *INO1* transcriptional memory are shared by diverse yeast genes.

Nuclear pore proteins also interact with metazoan genes to promote their transcription. Inhibiting histone deacetylase activity using trichostatin A in human cells leads to derepression of hundreds of genes, many of which physically interact with the NPC [8]. In *Drosophila*, nuclear pore proteins interact with the *hsp70* locus, the X chromosome in male flies [26]–[28], and genome-wide, thousands of genes [9]. However, in metazoans, some nuclear pore proteins localize both at the NPC and in the nucleoplasm and genes that interact with nuclear pore proteins can localize either at the nuclear periphery or in the nucleoplasm [16],[29]. In *Drosophila*, of the 18,878 genes that interact with the nuclear pore protein Nup98, 3,810 interacted exclusively with NPC-associated Nup98 and 11,307 interacted exclusively with nucleoplasmic Nup98 (GenBank Accession NP_651187.2) [9]. As in yeast, the interaction of genes with nuclear pore proteins also promotes transcription in flies [9],[16].

Here we sought to explore the role of nuclear pore interactions with genes in promoting transcriptional memory in humans. HeLa cells treated with IFN-γ show much faster and stronger expression of certain target genes if they have previously encountered IFN-γ [30]. This effect persists for up to four cell divisions (96 h), suggesting that it is epigenetically inherited through mitosis [31]. This phenomenon is also associated with changes in chromatin structure; dimethylated histone H3 lysine 4 (H3K4me2) remains associated with the promoter of the interferon-γ (IFN-γ)-inducible gene *HLA-DRA* for up to 96 h after treatment with IFN-γ [31]. Here, we have determined the scope of IFN-γ transcriptional memory in human cells and compared it with the molecular mechanisms of *INO1* transcriptional memory in yeast. Hundreds of the genes that are induced by IFN-γ exhibit transcriptional memory. Following expression, yeast and human genes that exhibit transcriptional memory are marked by H3K4me2 and associate with both a poised RNAPII and Nup100/Nup98 (GenBank Accession AAH12906.2) for up to four generations. Loss of Nup100 in yeast, or transient knockdown of Nup98 in HeLa cells, leads to loss of RNAPII and H3K4me2 from recently expressed promoters and a slower rate of reactivation of genes that exhibit memory. Thus, Nup100/Nup98 is required for epigenetic transcriptional memory, a mechanism conserved from yeast to humans.

## Results

### A Subset of Preinitiation Complex Components Interact with the *INO1* Promoter after Repression

**Figure 1:**
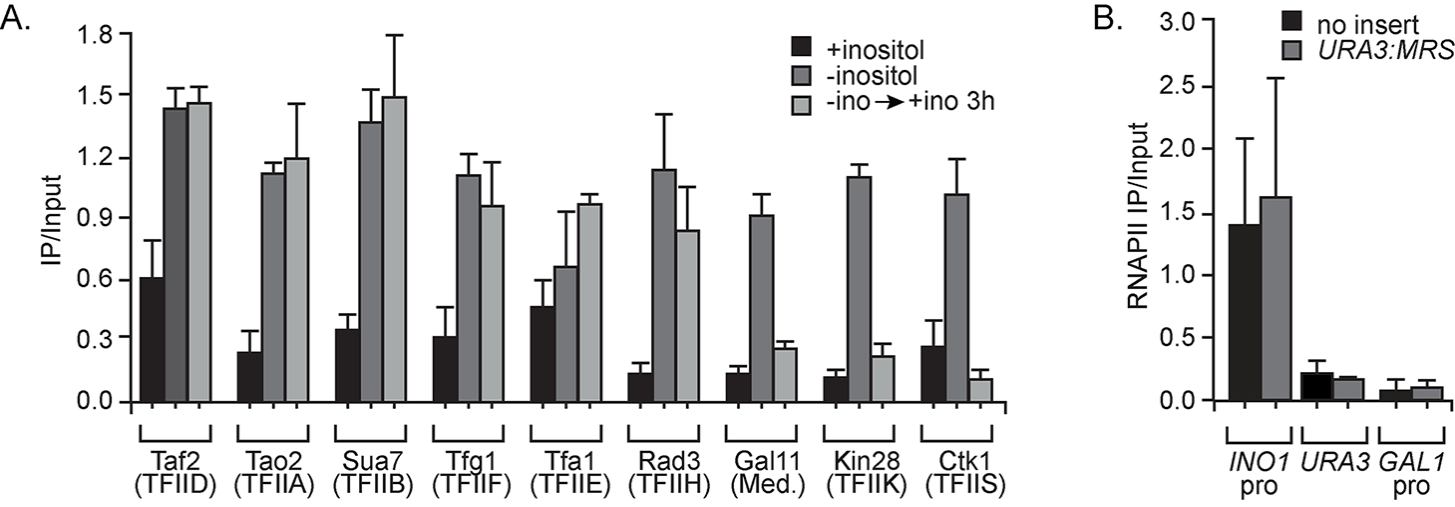
A subset of PIC components interacts with the *INO1* promoter after repression. (A) TAP-tagged strains were grown under long-term repressing (+inositol, black bars), activating (−inositol, dark grey bars), or recently repressed (−ino→+ino 3 h, light grey bars) conditions and processed for chromatin immunoprecipitation (ChIP). The recovery of the *INO1* promoter was quantified by qPCR relative to input. Individual tagged subunits and the PIC complex component are indicated. (B) Cells with (grey bars) or without (black bars) the MRS inserted beside *URA3* [18], grown in recently repressed conditions, were fixed and subjected to ChIP using anti-RNAPII (8WG16). The recovery of the *INO1* promoter or the indicated loci was quantified by qPCR relative to input. For all panels, error bars represent standard error of the mean from three experiments.

After yeast cells are shifted from medium lacking inositol into medium containing inositol, *INO1* transcription is rapidly repressed and the mRNA returns to baseline within ∼30 min [17],[18],[23]. RNAPII dissociates from the body of the gene after addition of inositol, but it remains associated with the *INO1* promoter for up to four generations after repression [17],[18],[23]. This form of RNAPII is unphosphorylated on the carboxy terminal domain (CTD) on serine 5 (associated with transcription initiation) and serine 2 (associated with transcription elongation), suggesting that it represents a preinitiation form [18]. To explore the nature of RNAPII that is associated with the recently repressed *INO1* promoter, we monitored the association of preinitiation complex (PIC) components before, during, and after expression of *INO1*. We performed ChIP using strains expressing Tandem Affinity Purification (TAP)-tagged components of TFIID, TFIIA, TFIIB, TFIIF, TFIIE, TFIIH, TFIIK, TFIIS, and Mediator from cells grown in long-term repressing (+inositol), activating (−inositol), or recently repressed (−ino→+ino, 3 h) conditions. None of these PIC components bound to the long-term repressed *INO1* promoter (Figure 1A). However, like RNAPII, the PIC components TFIID, TFIIA, TFIIB, TFIIF, TFIIE, and TFIIH bound to the promoter both when the gene was active and after repression (Figure 1A). In contrast, three PIC components interacted with the active promoter, but not the recently repressed promoter: the TFIIK component Kin28 (GenBank Accession CAA64904.1, the kinase that phosphorylates serine 5 on the CTD) [32],[33], the TFIIS component Ctk1 (GenBank Accession CAA81980.1, the kinase that phosphorylates serine 2 on the CTD) [34]–[36], and the Mediator component Gal11 (GenBank Accession CAA99056.1) [35],[36]. This is consistent with the conclusion that the RNAPII that binds to the recently repressed *INO1* promoter is not phosphorylated on Ser2 or Ser5 of the CTD [18]. We confirmed that Ser5 phosphorylated RNAPII did not remain associated with the *INO1* promoter after repression using a monoclonal antiphospho Ser5 CTD antibody (mAb 4h8; Figure S1). Together, these results suggest that a novel, partially assembled PIC associates with the recently repressed *INO1* promoter. Furthermore, binding of these components is not sufficient to induce transcription, suggesting that *INO1* reactivation is regulated by controlling the association of Mediator, TFIIK, and/or TFIIS.

### The MRS DNA Zip Code Is Not Sufficient to Cause RNAPII Binding

*INO1* transcriptional memory requires an 11 base pair *cis*-acting element called the MRS in the promoter [18]. Mutation of the MRS blocks interaction of recently repressed *INO1* with the NPC, incorporation of the histone variant H2A.Z, and binding of RNAPII to the recently repressed *INO1* promoter, resulting in a slower rate of reactivation of *INO1* [18]. When inserted at an ectopic locus, the MRS is sufficient to induce both H2A.Z incorporation and interaction with the NPC [18]. To test if the MRS was also sufficient to induce the association of a poised RNAPII at an ectopic locus, we inserted the MRS adjacent to the *URA3* locus (GenBank Accession AAB64498.1) [18] and performed ChIP for RNAPII. We fixed and harvested cells that had been shifted from activating to repressing conditions for 3 h so that we could simultaneously monitor the recovery of the endogenous *INO1* locus as an internal positive control. Although RNAPII associated with the recently repressed *INO1* promoter under these conditions, it did not associate with *URA3* or *URA3:MRS* or a negative control locus (the *GAL1* promoter; Figure 1B). Therefore, the MRS is not sufficient to recapitulate all facets of *INO1* transcriptional memory and assembly of the PIC requires other features of the promoter. This suggests that the interaction with the NPC and the incorporation of H2A.Z occur upstream of, and presumably promote, assembly of the PIC.

### The Human Gene *HLA-DRA* Exhibits Epigenetic Transcriptional Memory

**Figure 2:**
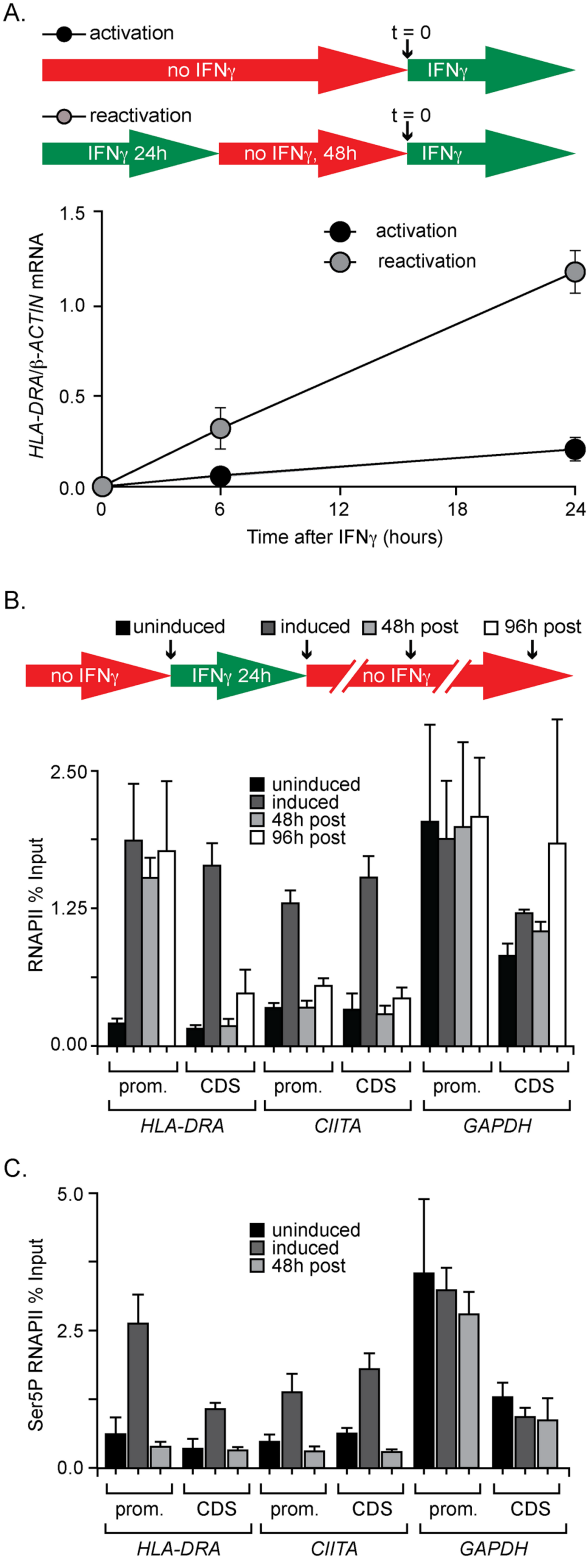
Human *HLA-DRA* exhibits epigenetic transcriptional memory. (A, Top) Schematic of activation and reactivation time courses. For activation, HeLa cells were mock treated for 72 h and then treated with IFN-γ. For reactivation, cells were first treated with IFN-γ for 24 h, washed and split into fresh medium, cultured for 48 h without IFN-γ, and then treated again with IFN-γ. (Bottom) RT qPCR on RNA harvested from cells at the indicated times during activation and reactivation. The levels of *HLA-DRA* mRNA were quantified relative to *β*-*ACTIN*. The positions of all qPCR products for human genes are shown in Figure S3. Error bars represent the standard error of the mean from five experiments. (B, Top) Schematic of treatment regime. (Bottom) Cells were fixed and harvested at the indicated times and ChIP was performed using anti-RNAPII (8WG16). The recovery of promoter and coding sequences for *HLA-DRA*, *CIITA*, and *GAPDH* was quantified by qPCR relative to input. (C) Cells were treated as in panel B, fixed, and ChIP was performed using anti-phospho-Ser5 CTD (4h8). The recovery of promoter and coding sequences for *HLA-DRA*, *CIITA*, and *GAPDH* was quantified by qPCR relative to input. For panels B and C, error bars represent standard error of the mean from three experiments.

The *HLA-DRA* gene in HeLa cells (GenBank Accession CAG33294.1, encoding the HLA class II histocompatibility antigen DRα chain) exhibits a form of transcriptional memory in response to IFN-γ. Cells previously treated with IFN-γ induce *HLA-DRA* more rapidly and more robustly in response to subsequent exposure to IFN-γ (Figure 2A) [31]. Not all IFN-γ-inducible genes behave this way; another IFN-γ-inducible gene, *CIITA* (GenBank Accession NP_000237.2), does not display transcriptional memory [31]. Similar to *INO1* transcriptional memory, this type of transcriptional memory is epigenetically inherited, persisting through at least four cell divisions in HeLa cells (96 h) [31]. These similarities led us to ask if these two systems utilize related molecular mechanisms.

We used ChIP to examine the association of RNAPII with the *HLA-DRA* promoter before (uninduced), during (induced), or at various times after treatment with IFN-γ [48 h (∼2 cell divisions) and 96 h (∼4 cell divisions) postinduction]. Prior to IFN-γ treatment, RNAPII was not associated with the promoter or the coding sequence of *HLA-DRA* and *CIITA* (Figure 2B). This is consistent with the undetectable levels of *HLA-DRA* and *CIITA* mRNA before IFN-γ treatment (Figure 2A). During IFN-γ treatment, RNAPII associated strongly with the promoter and the coding sequence of both *HLA-DRA* and *CIITA* (Figure 2B). After removing IFN-γ, RNAPII remained associated with the *HLA-DRA* promoter, but not the coding sequence, for up to 96 h (Figure 2B). RNAPII did not associate with *CIITA* after removing IFN-γ and the levels of RNAPII associated with *GAPDH* (GenBank Accession AAH83511.1) promoter and coding sequence were consistent under all three conditions (Figure 2B). Therefore, *HLA-DRA* memory correlates with persistent RNAPII binding to the previously induced promoter through at least four cell divisions.

Following treatment of cells with IFN-γ, the signaling and transcriptional response can persist even after washing, presumably because of persistent association of IFN-γ with the IFN-γ receptor and signaling through the JAK/STAT pathway (Figure S2A and B) [37]. For this reason, we trypsinized and split the cells after removing IFN-γ in all of our experiments, which leads to the levels of *HLA-DRA* mRNA returning to baseline levels within 6 h (Figure S2A). Likewise, treatment of HeLa cells with IFN-γ immediately after trypsinizing did not result in expression of *HLA-DRA* (Figure S2B), suggesting that trypsin digestion blocks IFN-γ signaling. Thus, the association of RNAPII with the *HLA-DRA* promoter is not due to persistent expression. This is consistent with loss of RNAPII from the *HLA-DRA* coding sequence after removing IFN-γ and splitting (Figure 2B).

We also tested if previous expression of *HLA-DRA* is necessary for transcriptional memory. Exposure of cells to IFN-γ for 2 h, followed by splitting the cells, does not result in significant *HLA-DRA* expression (Figure S2B). However, this brief exposure to IFN-γ is sufficient to induce a faster rate of reactivation 48 h later (∼2 cell divisions; Figure S1C). Thus, previous expression of *HLA-DRA* is not necessary to induce future transcriptional memory.

In metazoans, transcription is regulated both by blocking RNAPII recruitment and by blocking RNAPII elongation [38]–[42]. In the latter case, RNAPII binds to the promoter, initiates transcription, and then pauses at the 5′ end of the gene due to regulation by negative elongation factor (NELF; GenBank Accession AAI10499.1) and DRB sensitivity-inducing factor (DSIF; GenBank Accession BAA24075.1) [43]. This paused RNAPII is phosphorylated on Ser5 of the CTD, but unphosphorylated on Ser2 [38],[44],[45]. Transcription of such genes is stimulated by recruitment of the kinase P-TEFb, which phosphorylates Ser2 and allows elongation [46]. In yeast, ChIP using a monoclonal anti-phospho serine 5 antibody (mAb 4h8) recovers active, but not recently repressed *INO1* promoter (Figure S2). We performed ChIP using mAb 4h8 to ask if the RNAPII associated with the previously expressed *HLA-DRA* promoter is postinitiation or preinitiation. Ser5 phosphorylated RNAPII bound to the active *HLA-DRA* promoter in cells exposed to IFN-γ, but not with the previously expressed *HLA-DRA* promoter after removal of IFN-γ (Figure 2C). Therefore, similar to yeast *INO1*, the *HLA-DRA* promoter associates with a preinitiation form of RNAPII for several cell divisions after removing IFN-γ.

### Nuclear Pore Proteins Interact with Both Active and Previously Expressed *HLA-DRA*

**Figure 3:**
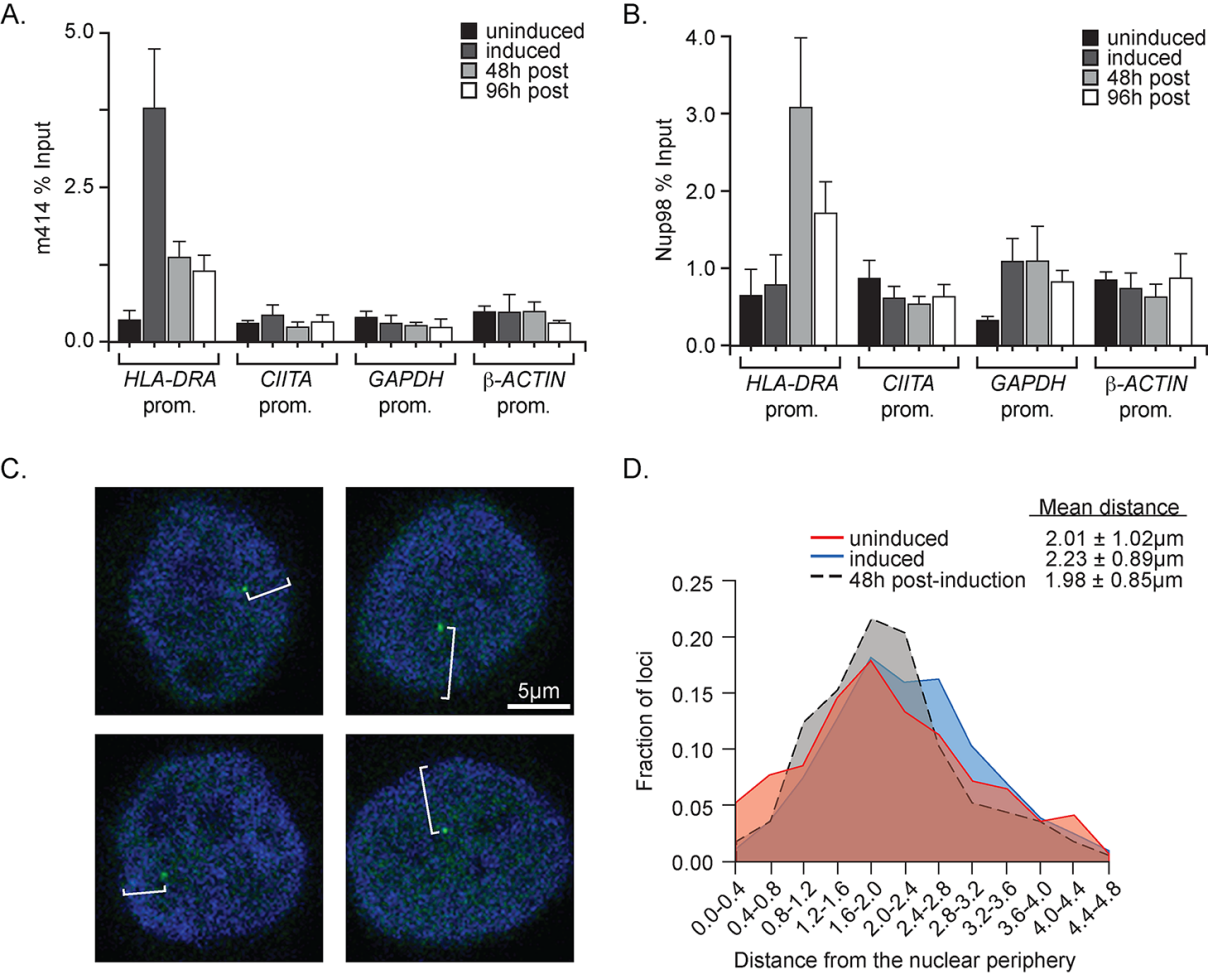
*HLA-DRA* interacts with nuclear pore proteins in the nucleoplasm. Cells were treated as in Figure 2B, fixed, and ChIP was performed using m414 (A) or anti-Nup98 (B). The recovery of the promoters of *HLA-DRA*, *CIITA*, *GAPDH*, and *β*-*ACTIN* was quantified by qPCR relative to input. Error bars represent standard error of the mean from three experiments. (C) Representative confocal *z* slices from DNA-FISH against HeLa cells using probes from a BAC overlapping the *HLA-DRA* gene. *HLA-DRA* signal is green, and Hoechst staining is blue. Each of the three *HLA-DRA* loci was scored independently from individual *z* slices. The distances to the nuclear periphery for each cell are indicated. Scale bar, 5 µm. (D) Distribution of DNA-FISH measurements from FISH focus to the edge of nuclear staining for ∼300 foci. Distances were binned into 0.4 µm bins and the distribution of distances within the population is plotted. Red, uninduced cells; blue, cells treated with IFN-γ for 24 h; black, 48 h after removal of IFN-γ. The mean distances to the nuclear periphery ± standard deviation are reported for each distribution.

In yeast, distinct Nups interact with active [11] and recently repressed *INO1* [18]. To test if *HLA-DRA* interacts with Nups, we performed ChIP using the mAb 414 monoclonal antibody, which recognizes Phe-x-Phe-Gly repeats present in several nuclear pore proteins [47],[48]. We observed strong interaction of Phe-x-Phe-Gly repeat proteins with the active *HLA-DRA* promoter and a weaker interaction after removing IFN-γ (Figure 3A). This pattern was very similar to the interaction of the Phe-x-Phe-Gly repeat protein Nup2 (GenBank Accession AAB67259.1) with the *INO1* promoter in yeast [18]. The interaction was also specific; mAb 414 did not recover the *HLA-DRA* coding sequence (not shown) nor the promoters of *CIITA*, *GAPDH*, and *β*-*ACTIN* (Figure 3A). This suggests that Phe-x-Phe-Gly Nups interact with the active and recently expressed *HLA-DRA* promoter.

The yeast nuclear pore protein Nup100 interacts with the *INO1* promoter specifically after repression, and not during activation [18]. We performed ChIP using an antibody against Nup98, a human homologue of Nup100 [1], and analyzed the interaction with the *HLA-DRA* promoter. Nup98 did not interact with the *HLA-DRA* promoter before or during IFN-γ treatment (Figure 3B). However, for up to 96 h (∼4 cell divisions) after removal of IFN-γ, we observed a clear and specific association of Nup98 with the *HLA-DRA* promoter. Therefore, similar to the specific interaction of Nup100 with the recently repressed *INO1* promoter, Nup98 interacts specifically with the recently expressed *HLA-DRA* promoter.

### *HLA-DRA* Interacts with Nups in the Nucleoplasm

In *Drosophila*, genes interact with Nups both at the NPC and in the nucleoplasm [9],[16]. In particular, Nup98 has been shown to localize both at the NPC and the nuclear periphery [49]. To test if the *HLA-DRA* interaction with Nup98 occurs at the NPC, we localized the *HLA-DRA* gene with respect to the nuclear periphery using DNA fluorescence in situ hybridization (FISH). Cells were fixed before (uninduced), during (induced), or after (48 h postinduction) treatment with IFN-γ and processed for DNA-FISH using fluorescent probes generated by nick translation of bacterial artificial chromosomes (BACs). Using confocal microscopy, we measured the distance from the individual *HLA-DRA* foci to the edge of the Hoescht fluorescence within individual *z* slices (Figure 3C). The distribution of these distances was plotted for ∼300 foci. Under all three conditions, the *HLA-DRA* gene localized in the nucleoplasm (Figure 3D). Active *HLA-DRA* was somewhat more nucleoplasmic than the preinduced *HLA-DRA* (*p* = 0.004, two-tailed *t* test) or postinduced *HLA-DRA* (*p* = 0.001). The position of *CIITA* with respect to the nuclear periphery did not change under these conditions (Figure S4). This suggests that *HLA-DRA* interacts with Nups away from the NPC, in the nucleoplasm.

### The Global Extent of IFN-γ-Induced Transcriptional Memory

**Figure 4:**
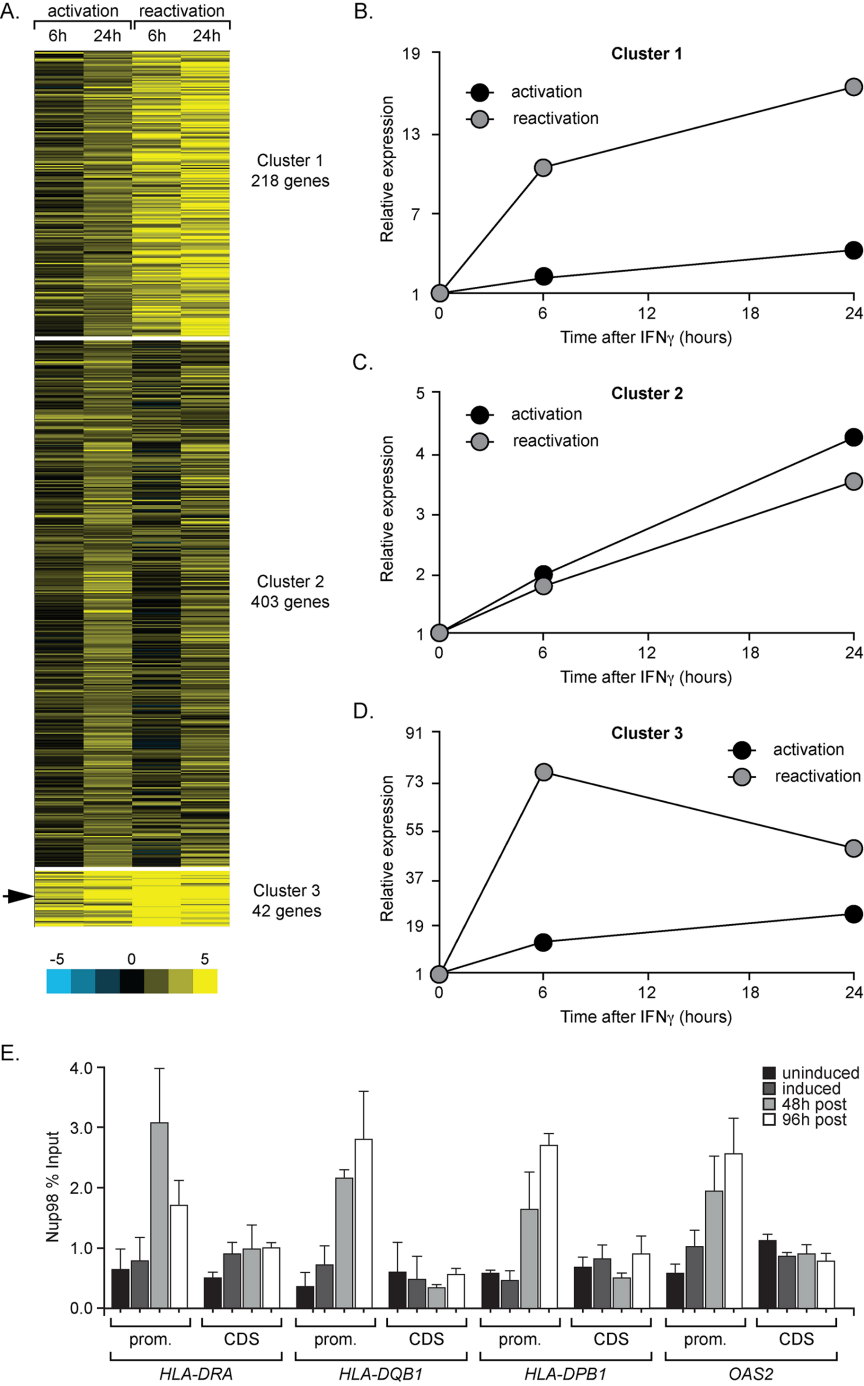
Many human genes exhibit IFN-γ memory. (A) The expression change of 664 IFN-γ-inducible genes during activation and reactivation. Shown is the log_2_ change in expression from Agilent 128×135K expression microarrays, relative to 0 h (scale below). These genes were subjected to *k* means clustering to identify three distinct clusters. Cluster 3 includes *HLA-DRA* (arrow). (B–D) The average change in expression, relative to time = 0, of genes from each cluster during activation and reactivation. (E) Cells were treated as in Figure 2B, fixed, and ChIP was performed with Nup98 antisera. The recovery of the promoters and coding sequences of candidate genes from Clusters 1 (*HLA-DQB1*) and 3 (*HLA-DRA*, *HLA-DPB1*, and *OAS2*) was quantified relative to input by qPCR. Error bars represent the standard error of the mean from three experiments.

To probe the generality of IFN-γ-induced transcriptional memory throughout the human genome, we performed expression microarrays on cDNA from cells treated with IFN-γ. We compared samples from time points either during initial activation or during reactivation after 48 h without IFN-γ (∼2 cell divisions, as in Figure 2A). The log_2_ ratios relative to the initial time point (0 h) were calculated by averaging between replicates and, for genes with multiple probes, between probes. Based on the initial activation after addition of IFN-γ, we identified a subset of 664 genes that were induced ≥2 fold between 6 h and 24 h (Table S1). Gene ontology (GO) analysis revealed that this subset of genes was highly enriched for terms related to innate immunity: “regulation of immune system process” (*p* = 7.76×10^−23^), “response to interferon gamma” (*p* = 3.99×10^−18^), and “response to cytokine stimulus” (*p* = 1.16×10^−17^; Table S2) [50]. We then used *k* means clustering to organize this subset into clusters on the basis of their behaviors during activation and reactivation (Figure 4A and Table S1).

Cluster 1 includes 218 genes that were modestly induced during activation and more strongly induced during reactivation (see average behavior in Figure 4B). This cluster was strongly enriched for genes involved in inflammation and genes regulated by infection (Table S3). Cluster 2 includes 403 genes that were induced equivalently during activation and reactivation (Figure 4C). This cluster was enriched for GO terms associated with innate immunity (Table S4). Cluster 3 includes 42 of the most strongly induced genes that were, nonetheless, induced more rapidly during reactivation (Figure 4D). This cluster includes *HLA-DRA* (Figure 4A, arrow) and was highly enriched for GO terms associated with cytokine signaling generally and IFN-γ signaling in particular (Table S5).

Many of the genes in Cluster 1 and most of the genes in Cluster 3 displayed mRNA profiles consistent with transcriptional memory. We analyzed genes from each of these clusters by RT qPCR to confirm this behavior. The Cluster 1 gene *HLA-DQB1* (GenBank Accession AAA59770.1) and the Cluster 3 genes *HLA-DPB1* (GenBank Accession AAA59837.1) and *OAS2* (GenBank Accession AAH10625.1) displayed significantly faster and/or stronger activation in cells previously exposed to IFN-γ (Figure S5B–D). *HLA-DQB1* and *HLA-DPB1* encode the HLA class II histocompatibility antigen DQα and DPβ chains, respectively, and *OAS2* encodes a 2′-5′-oligoadenylate synthetase [51],[52]. However, the clustering algorithm did not result in perfect segregation of genes with memory from genes without memory; the gene *CIITA*, which does not exhibit obvious transcriptional memory (Figure S5A) [31], was within Cluster 1. Regardless, these results suggest that a large subset of the genes induced by IFN-γ exhibit stronger or more rapid induction in response to IFN-γ if the cells have been previously exposed to IFN-γ.

To test if other genes that exhibit memory are regulated by the same mechanism as *HLA-DRA*, we used ChIP against RNAPII and Nup98 before, during, and after IFN-γ treatment. Both RNAPII (Figure S5E) and Nup98 (Figure 4E) bound to the promoters of *HLA-DQB1*, *HLA-DPB1*, and *OAS2* for up to 96 h (∼4 cell divisions) after removing IFN-γ. Neither RNAPII (Figure S5E) nor Nup98 (Figure 4C) bound to the coding sequences of these genes after removal of IFN-γ. Therefore, the interaction of RNAPII and Nup98 with recently expressed promoters is a general feature of IFN-γ memory.

### Transcriptional Memory in Yeast and Humans Is Associated with Changes in Histone H3 Methylation

Transcriptional memory in yeast and humans is associated with changes in chromatin. In yeast, the histone variant H2A.Z is incorporated into a single nucleosome in the *INO1* promoter after repression and loss of H2A.Z blocks *INO1* transcriptional memory [17],[18]. It is unclear if H2A.Z is involved in IFN-γ transcriptional memory; *HLA-DRA* memory is associated with a very slight increase in H2A.Z incorporation into the promoter after removal of IFN-γ (Figure S5F). However, *HLA-DRA* transcriptional memory is associated with persistent dimethylation of histone H3 lysine 4 (H3K4me2) [31]. Histone H3 in nucleosomes at the 5′ end of actively transcribed genes are trimethylated on lysine 4 (H3K4me3) [53],[54]. Whereas the H3K4me3 mark is lost from the promoter of *HLA-DRA* after removal of IFN-γ, the H3K4me2 mark remains (Figure S6A and B) [31]. In contrast, both marks are lost from the *CIITA* promoter after removing IFN-γ (Figure S6A and B) [31].

**Figure 5:**
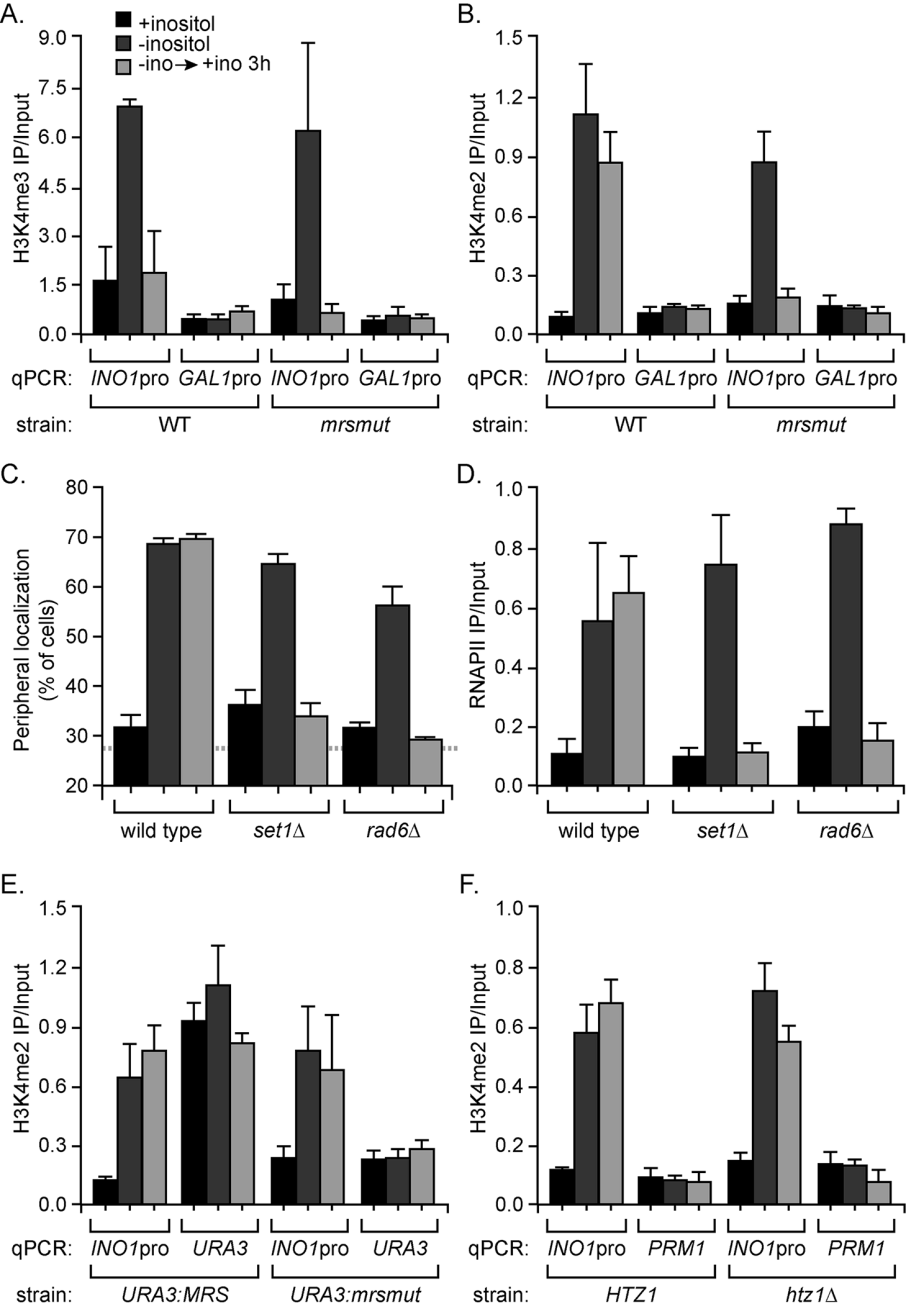
H3K4 dimethylation is necessary for *INO1* transcriptional memory. For panels A–F, cells were grown under long-term repressing (+inositol, black bars), activating (−inositol, dark grey bars), or recently repressed (−ino→+ino 3 h, light grey bars) conditions. For panels A, B, D, E, and F, the recovery of the *INO1* promoter was quantified by qPCR relative to input. For all panels, error bars represent the standard error of the mean from three experiments. Wild-type and *mrs* mutant strains were fixed and subjected to ChIP using anti-H3K4me3 (A) or anti-H3K4me2 (B). The *GAL1-10* promoter served as a negative control in panels A and B. (C) *INO1* peripheral localization was quantified by localizing the LacO array bound to LacI-GFP with respect to the nuclear envelope, stained against Sec63-myc [91]. The blue, hatched line represents the baseline for peripheral localization in this assay [12]. Three biological replicates of 30–50 cells were scored. (D) Wild-type, *set1*Δ, and *rad6*Δ cells were fixed and subjected to ChIP using anti-RNAPII (8WG16). (E) The MRS or *mrs mutant* was inserted at *URA3* [15], and cells were fixed and subjected to ChIP using anti-H3K4me2. The recovery of the *INO1* promoter or the insertion site at *URA3* was quantified by qPCR relative to input. (F) Wild-type and *htz1*Δ cells were fixed and subjected to ChIP using anti-H3K4me2.

To test if H3K4me2 was associated with *INO1* transcriptional memory in yeast, we examined the association of H3K4me3 and H3K4me2 with the *INO1* promoter under long-term repressing, activating, or recently repressed conditions. As a control, we used a strain in which the MRS had been mutated and *INO1* transcriptional memory is blocked [18]. H3K4me3 was only associated with the active *INO1* promoter (Figure 5A). However, H3K4me2 was associated with both the active and recently repressed *INO1* promoters (Figure 5B). The persistence of H3K4me2 after repression required the MRS (Figure 5B). Therefore, *INO1* transcriptional memory is also associated with dimethylation of H3K4.

### Dimethylation of Histone H3 Lysine 4 Is Required for *INO1* Transcriptional Memory

We next asked if the machinery responsible for methylation of H3K4 was required for other aspects of *INO1* transcriptional memory; namely, poised RNAPII association and localization at the nuclear periphery after repression. In yeast strains lacking either the histone methyltransferase Set1 (GenBank Accession AAB68867.1) or E2 ubiquitin-conjugating enzyme Rad6 (GenBank Accession CAA96761.1), all di- and tri-methylation of H3K4 is lost [55]. Loss of these enzymes did not affect the localization of active *INO1* to the nuclear periphery (Figure 5C) or interaction of RNAPII with active *INO1* (Figure 5D). However, loss of either Set1 or Rad6 specifically disrupted both localization of recently repressed *INO1* at the nuclear periphery (Figure 5C) and RNAPII binding to the recently repressed *INO1* promoter (Figure 5D). This suggests that H3K4me2 at the *INO1* promoter is required for *INO1* transcriptional memory.

The MRS is necessary for both incorporation of H2A.Z [18] and the persistent dimethylation of H3K4 (Figure 5B) at the recently repressed *INO1* promoter. Integration of the MRS at ectopic sites is sufficient to induce H2A.Z deposition [18]. Therefore, we asked if the MRS was also sufficient to induce dimethylation of H3K4 at an ectopic locus. We performed ChIP against H3K4me2 in a strain in which either the MRS or the nonfunctional *mrs* mutant was integrated beside *URA3* [18]. We observed a robust signal for H3K4me2 associated with *URA3:MRS* but not with *URA3:mrsmut* (Figure 5E) or a control locus (the coding sequence of the repressed gene *PRM1*; not shown). We also observed a small but reproducible increase in H3K4me3 at *URA3:MRS* compared with *URA3:mrsmut*, although this level was significantly lower than the level associated with the active *INO1* promoter (Figure S6C). Therefore, the MRS is sufficient to induce both H2A.Z incorporation and dimethylation of H3K4, recapitulating the chromatin changes associated with *INO1* transcriptional memory.

Loss of H2A.Z or H3K4 dimethylation leads to loss of *INO1* transcriptional memory and both of these modifications require the MRS (Figure 5) [17],[18]. We next asked if the methylation of H3K4 at the recently repressed *INO1* promoter required H2A.Z. In wild-type and *htz1*Δ strains, we observed similar levels of H3K4me3 (Figure S6D) and H3K4me2 (Figure 5F) at the active and recently repressed *INO1* promoter. Therefore, dimethylation of H3K4 at the recently repressed *INO1* promoter requires the MRS, but not H2A.Z. Furthermore, although strains lacking H2A.Z show slower *INO1* reactivation kinetics, loss of peripheral localization of recently repressed *INO1*, and loss of RNAPII from the *INO1* promoter after repression [18], the *INO1* promoter is still marked by H3K4me2. This suggests that dimethylation of H3K4 occurs upstream of, or independent of, H2A.Z deposition to promote *INO1* transcriptional memory.

### Nup98 Is Necessary for IFN-γ Transcriptional Memory

**Figure 6:**
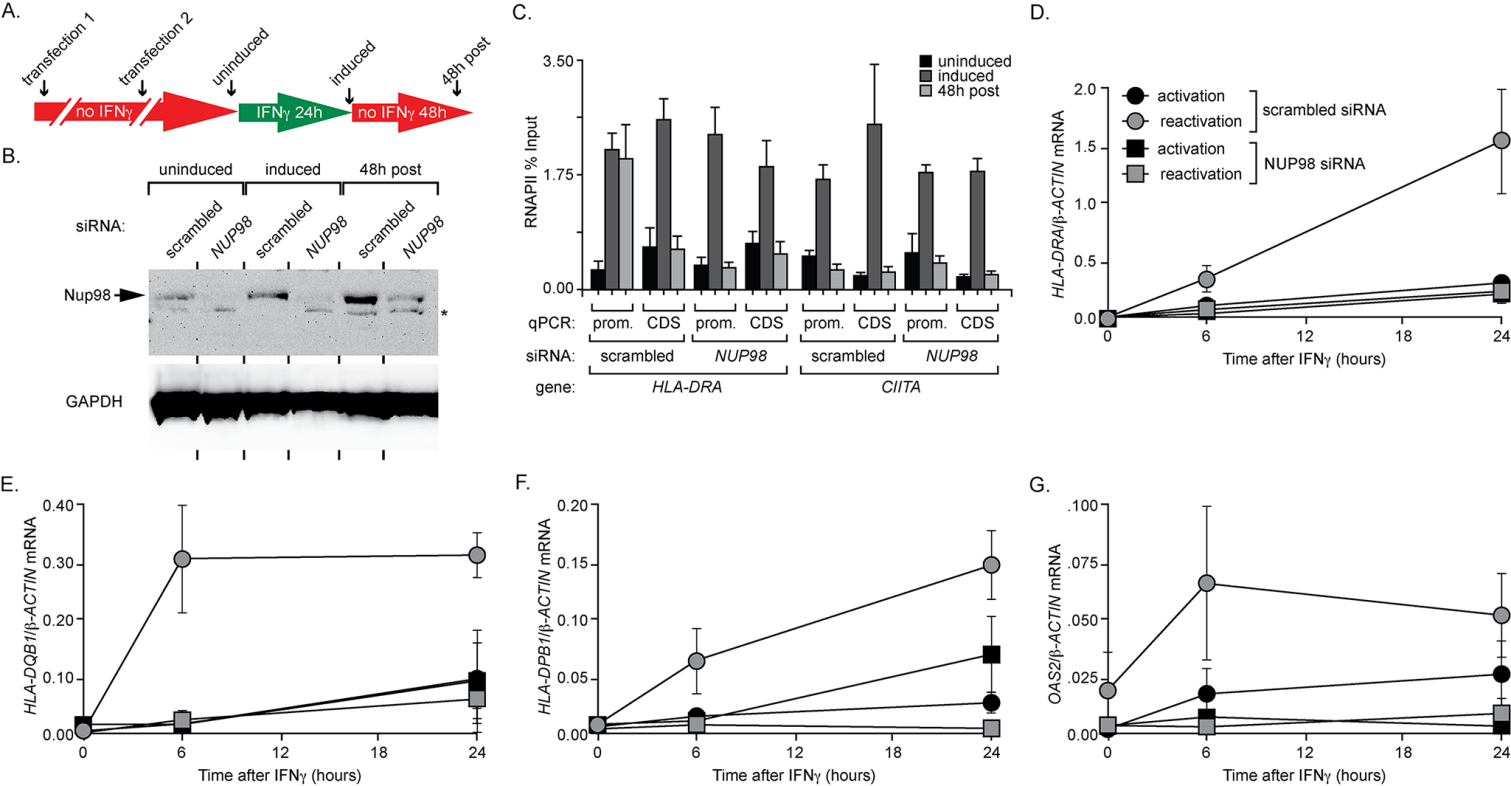
Nup98 is necessary for IFN-γ memory. (A) Schematic of Nup98 knockdown strategy: on the 2 d prior to IFN-γ treatment, cells were serially transfected for 6 h with pools of either scrambled siRNA or *NUP98* siRNA. (B) Lysates from cells treated as shown in (A) was subjected to Western blot. GAPDH was used as a loading control, and the asterisk indicates nonspecific background for Nup98 antibody. (C) ChIP from cells treated as shown in panel A using anti-RNAPII (8WG16). The promoters and coding sequences of *HLA-DRA* and *CIITA* were quantified by qPCR. (D–G) Nup98 was knocked down as indicated in panel A, and cells were then subjected to activation and reactivation regimes as in Figure 2A. Legend in panel D applies to panels D–G. The mRNA levels for *HLA-DRA* (D), *HLA-DQB1* (E), *HLA-DPB1* (F), and *OAS2* (G) were quantified by RT qPCR during activation and reactivation relative to *β-ACTIN*. For all panels, error bars represent the standard error of the mean for three experiments.

*INO1* transcriptional memory requires Nup100 for both rapid reactivation and RNAPII association after repression [18]. Because we observed a specific physical interaction of Nup98 with the previously induced *HLA-DRA* promoter, we asked if Nup98 was required for IFN-γ transcriptional memory. We used transient siRNA knockdown to reduce the levels of Nup98 prior to expression and ChIP analysis (schematized in Figure 6A). Transient knockdown reduced Nup98 during the time course of the experiment; 5 d after transfection, Nup98 protein levels were still reduced, while at earlier times Nup98 was not detected (Figure 6B). Both nuclear pore-associated Nup98 and nucleoplasmic Nup98 were depleted by this treatment (Figure S7). We did not observe a significant change in the growth rate or morphology of the cells subjected to this treatment.

Knockdown of Nup98 had no apparent effect on RNAPII binding to active *HLA-DRA* (Figure 6C), on the rate of initial activation of *HLA-DRA* (Figure 6D), or on the association of RNAPII with the *CIITA* gene (Figure 6C). However, knockdown of Nup98 blocked binding of RNAPII to the *HLA-DRA* promoter following removal of IFN-γ (Figure 6C) and dramatically reduced the rate of reactivation of *HLA-DRA* (Figure 6D). This suggests that Nup98 is required for *HLA-DRA* transcriptional memory.

Because Nup98 bound to the promoters of the Cluster 1 gene *HLA-DQB1* and the Cluster 3 genes *HLA-DPB1* and *OAS2* after removal of IFN-γ (Figure 4E), we tested the effect of Nup98 knockdown on the transcriptional memory of these genes. Transient knockdown of Nup98 reduced the rate of reactivation of all three genes (Figure 6E, F, and G). In the cases of *HLA-DQB1* and *OAS2*, this effect was specific for reactivation. However, in the case of *HLA-DPB1*, we also observed a slower rate of activation (Figure 6F). Therefore, knockdown of Nup98 affects the transcriptional memory of several human genes, although this effect may not be memory-specific in all cases.

**Figure 7:**
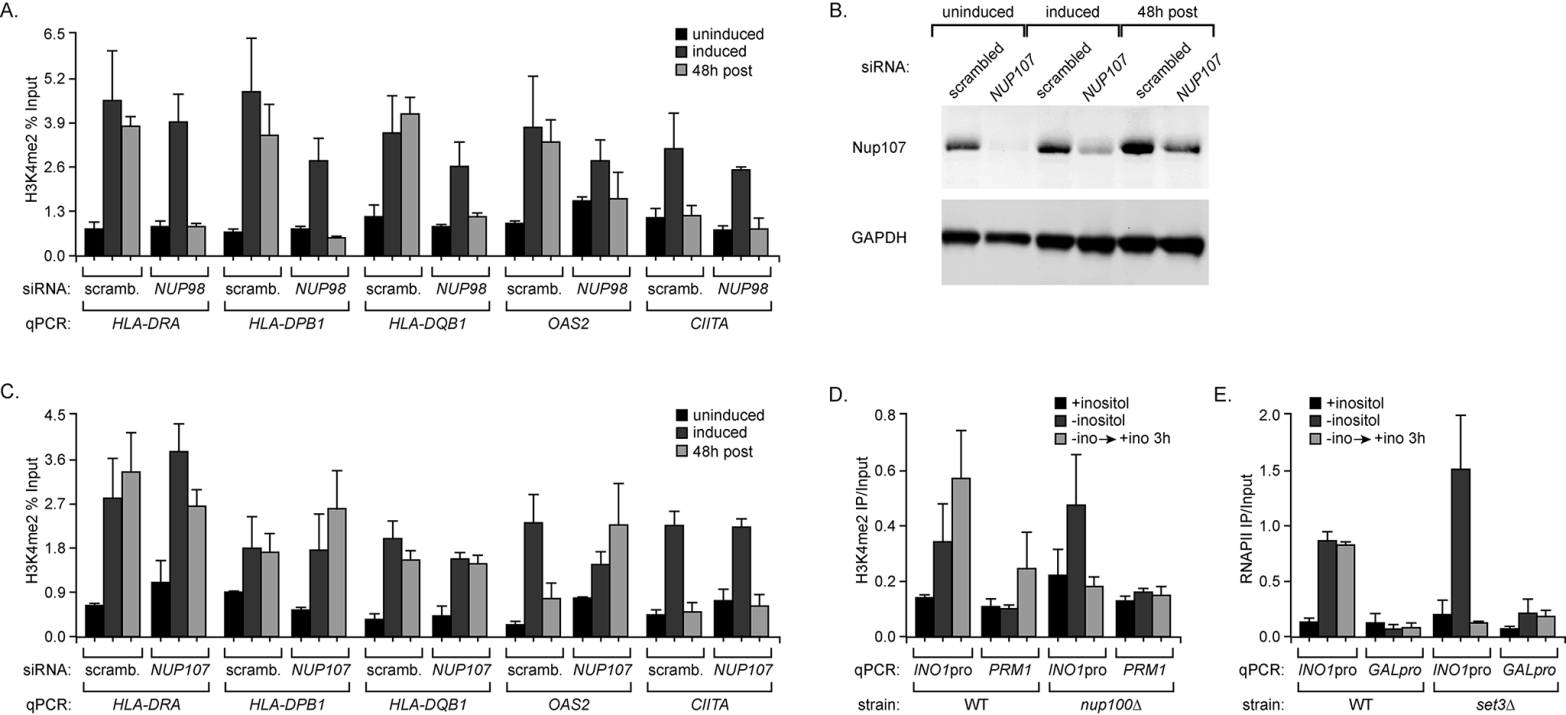
Nup98 and Nup100 are required for posttranscriptional dimethylation of H3K4 in human and yeast cells. (A) Nup98 knockdown was performed as schematized in Figure 6A, and cells were cultured without IFN-γ (uninduced, black), with IFN-γ for 24 h (induced, dark grey), or with IFN-γ for 24 h and then without IFN-γ for 48 h (48 h post, light grey). Cells were fixed and ChIP was performed against H3K4me2. Enrichment of indicated promoters was quantified relative to the input fraction using qPCR. (B) Western blots against Nup107 and GAPDH of lysates from cells treated with siRNA pools against *NUP107* as schematized in Figure 6A. (C) ChIP against H3K4me2 from cells knocked down for Nup107, quantified as in panel A. (D) Wild-type and *nup100*Δ cells grown in repressing (+inositol, black), activating (−inositol, dark grey), and recently repressed conditions (−ino→+ino 3 h, light grey) were subjected to ChIP against H3K4me2. Enrichment of the *INO1* promoter and the *PRM1* coding sequence were quantified by qPCR. (E) Wild-type and *set3*Δ mutant strains grown in repressing, activating, and recently repressed conditions were subjected to ChIP against RNAPII. Recovery of the *INO1* promoter or the *GAL1* promoter was quantified by qPCR. For all panels, error bars represent the standard error of the mean for three experiments.

To explore the role of Nup98 in regulating chromatin structure, we asked if the dimethylation of H3K4 associated with transcriptional memory required Nup98. We performed ChIP against H3K4me2 in cells knocked down for Nup98 and quantified the enrichment of this mark at the promoters of *HLA-DRA*, *HLA-DPB1*, *HLA-DQB1*, *OAS2*, and *CIITA* (Figure 7A). We observed H3K4me2 at the recently expressed promoters of *HLA-DRA*, *HLA-DPB1*, *HLA-DQB1*, and *OAS2*, and this mark was lost when Nup98 was knocked down (Figure 7A). Consistent with the impaired activation of *HLA-DPB1* in the absence of Nup98 (Figure 6G), we also observed a slight decrease in the level of H3K4me2 associated with the active *HLA-DPB1* promoter when Nup98 was knocked down (Figure 7A). Therefore, Nup98 is required for dimethylation of H3K4 at the promoters of genes with transcriptional memory.

To confirm that the effects of *NUP98* knockdown are specific, we tested the effect of knockdown of *NUP107*, a component of the core channel of the NPC [56], on H3K4 dimethylation of IFN-γ-inducible promoters. Using the same strategy to knockdown Nup107, the protein was effectively depleted (Figure 7B). H3K4me2 levels associated with the promoters of *HLA-DRA*, *HLA-DQB1*, *HLA-DPB1*, and *OAS2* after removal of IFN-γ remained high in the absence of Nup107 (Figure 7C). Therefore, the effects of Nup98 knockdown on transcriptional memory were specific.

We next asked if dimethylation of H3K4 at the recently repressed *INO1* promoter requires yeast Nup100. We grew wild-type and *nup100*Δ yeast strains in repressing, activating, and recently repressed conditions and performed ChIP against H3K4me2 and H3K4me3 (Figure 7D and Figure S8). Cells lacking Nup100 exhibited normal trimethylation (Figure S8) and dimethylation of H3K4 at the active *INO1* promoter but did not maintain H3K4me2 at the recently repressed *INO1* promoter (Figure 7D). Therefore, in yeast and human cells, Nup98/Nup100 is required for maintenance of histone H3 dimethylation during transcriptional memory.

Dimethylation of H3K4 is generally associated with the 5′ coding sequences of actively transcribed genes [57]. However, H3K3me2 of promoter regions, often associated with ncRNAs, leads to recruitment of the Set3 histone deacetylase complex (Set3C) and transcriptional repression [58]. We wondered if Set3C had any role in *INO1* transcriptional memory. This is somewhat complicated by the poor expression of *INO1* in mutants lacking Set3 (GenBank Accession EEU07596.1), suggesting that Set3 might have multiple effects on *INO1* expression [59]. However, we tested if Set3 plays a role in binding of RNAPII to the recently repressed *INO1* promoter. In mutant strains lacking Set3, RNAPII failed to remain associated with the *INO1* promoter after repression (Figure 7E). This suggests that recognition of H3K4me2 by Set3 is required for *INO1* transcriptional memory.

## Discussion

Here we further define the molecular mechanism of *INO1* transcriptional memory in yeast and demonstrate that aspects of this mechanism are conserved in human cells. Despite over a billion years of evolutionary time [60], in both systems, transcriptional memory requires the interaction of the nuclear pore protein Nup98/Nup100 with recently expressed promoters. This interaction leads to (1) dimethylation of histone H3 lysine 4 in promoter nucleosomes, (2) binding of a preinitiation form of RNAPII, and (3) faster reactivation. Loss of Nup98 in human cells and Nup100 in yeast leads to loss of H3K4me2 and RNAPII from the recently expressed promoters and a slower rate of reactivation. Therefore, Nup98/Nup100-dependent epigenetic transcriptional memory is a conserved mechanism of transcriptional regulation in both unicellular and multicellular organisms.

### Transcriptional Memory Regulates Transcription Initiation

Our work suggests that transcription can be regulated at three distinct stages: RNAPII recruitment, transcription initiation, and transcription elongation. In yeast, the primary mechanism by which transcription is regulated is through recruitment of RNAPII/PIC to the promoter and inducible genes tend to be devoid of RNAPII when uninduced or repressed [61]–[64]. However, under certain circumstances, a preinitiation form of RNAPII can associate with inactive yeast promoters. For example, in stationary phase cells, unphosphorylated RNAPII is associated with hundreds of inactive promoters, and this has been suggested to poise these genes for future activation [65]. Likewise, in metazoans, transcription can be regulated both at the level of PIC assembly and after initiation, at the level of transcription elongation [38],[44],[66]–[68]. Promoter-proximal pausing requires NELF and DSIF and is relieved by recruitment of pTEF-b [46],[69],[70]. Brewer's yeast lacks a homologue of NELF, and there is no conclusive evidence for RNAPII pausing [71]. Therefore, promoter-proximal pausing may be a metazoan-specific form of regulation [64].

Our results suggest that, for certain genes, the mechanism of regulation depends on the history of the cells. Transcription of such genes is regulated by either preventing RNAPII/PIC recruitment (under long-term repressing conditions) or allowing RNAPII/PIC recruitment but preventing transcription initiation (under recently repressed conditions). In the case of the *INO1* gene, whereas none of the PIC components bound to the long-term repressed *INO1* promoter, most of them bound to the recently repressed *INO1* promoter (Figure 1). This form of PIC is distinct from the PIC that associates with active *INO1*: TFIIK, Mediator, and TFIIS are absent and RNAPII remains unphosphorylated and fails to initiate. Thus, while the regulation of long-term repressed *INO1* prevents binding of RNAPII and the rest of the PIC, the regulation of recently repressed *INO1* occurs at a subsequent step. This suggests that the rate-limiting step in derepression is different for long-term repressed *INO1* and recently repressed *INO1*.

### Transcriptional Memory Is Epigenetically Inherited and Involves Histone Methylation

In both yeast and humans, transcriptional memory is inherited through cell division. In the case of yeast, the *INO1* gene remains at the nuclear periphery, associated with the NPC for ∼3–4 generations (≥6 h) in both the mother and daughter cells [17]. Likewise, RNAPII remains associated with the *INO1* promoter over the same number of generations [18]. HeLa cells exposed to IFN-γ exhibit faster and more robust activation of IFN-γ-inducible genes for up to ∼4 generations (96 h) after removing IFN-γ [31]. Binding of RNAPII and Nup98 to, and dimethylation of histone H3 lysine 4 over, the promoters of genes exhibiting transcriptional memory persists over the same number of generations. Therefore, the poised, preinitiation state is heritable through several generations, suggesting that it represents an epigenetic state.

Changes in chromatin composition and modification are necessary for transcriptional memory and presumably allow binding of RNAPII/PIC to recently expressed promoters in yeast and humans. For genes that display transcriptional memory in both yeast and humans, histone H3 within promoter nucleosomes was unmethylated on lysine 4 prior to induction (Figures 5 and 7). After expression, histone H3 within promoter nucleosomes was dimethylated on lysine 4 (H3K4me2; Figures 5 and 7) [31]. In human cells, this correlates with lower nucleosome occupancy of the recently expressed *HLA-DRA* promoter compared with the uninduced promoter [31]. In yeast, a *cis-*acting element necessary for *INO1* transcriptional memory (the MRS) was necessary for dimethylation of H3K4 after repression and insertion of the MRS at an ectopic locus is sufficient to induce both H2A.Z incorporation [18] and dimethylation of H3K4 (Figure 5). Finally, loss of the enzymes responsible for H3K4 methylation (Set1 or Rad6; Figure 5) or recognition of H3K4me2 (Set3; Figure 7) led to loss of *INO1* transcriptional memory. Although it is still formally possible that Set1 methylates another protein required for transcriptional memory, the connection to the *cis-*acting MRS element and the requirement for Rad6 and Set3 (Figure 7) suggest that Set1 methylation of H3K4 is required for transcriptional memory.

### Nuclear Pore Proteins Affect Chromatin Structure and Promote Transcriptional Memory

Binding of nuclear pore proteins to the promoters of genes impacts both their transcription and their epigenetic regulation. In yeast and *Drosophila*, nuclear pore proteins interact with the promoters of active genes and this interaction is required for their full expression [9],[12]–[16]. We have found another role for these interactions. The yeast nuclear pore protein Nup100 is required for *INO1* transcriptional memory and the homologous human protein Nup98 is required for IFN-γ-mediated memory.

One complication of any experiment manipulating Nup98 levels is that both Nup98 and Nup96 are generated from a single transcript [72]. After nuclear import, this protein undergoes autocatalytic cleavage, producing two proteins [73]. Indeed, knockdown of Nup98 also leads to depletion of Nup96 (Figure S9). Therefore, our results raise the possibility that both Nup98 and Nup96 impact transcriptional memory, especially since Nup96 has been implicated in promoting expression of interferon-responsive genes in mouse [74] and the Nup96 homologue Nup145C (GenBank Accession P49687) is required for localization of recently repressed *INO1* at the nuclear periphery in yeast [18]. Both Nup96 and Nup98 localize in the nucleoplasm and at the NPC [49],[75]. Our data suggest that Nup98 plays a direct and specific role: Nup98 binds to the promoters of genes that exhibit transcriptional memory specifically after removal of IFN-γ and knockdown affects reactivation rate without affecting activation rate. Also, although loss of Nup96 is lethal [74],[76], we did not observe a strong defect in the growth of cells transiently knocked down for Nup98, suggesting that Nup96 function was not completely depleted. And finally, knockdown of Nup107, another component of the same subcomplex of the NPC as Nup96, had no effect on H3K4 dimethylation of promoters of primed genes. We conclude that our data support a role for Nup98, and potentially Nup96, in transcriptional memory. Future work will be required to separate these two roles.

Phe-x-Phe-Gly repeat proteins that are recognized by mAb 414 interact with the promoter of both active *HLA-DRA* and recently expressed *HLA-DRA*. Nup98, which possesses related repeated motif (Gly-Leu-Phe-Gly), interacts specifically with the promoter of recently expressed *HLA-DRA*. This suggests that active *HLA-DRA* and recently expressed *HLA-DRA* interact with two distinct sets of nuclear pore proteins. This conclusion is very similar to what we have observed for *INO1*; active *INO1* and recently repressed *INO1* interact with different Nups, and localization of active and recently repressed *INO1* at the nuclear periphery requires different Nups and different DNA elements [18]. The interaction of active and recently expressed *HLA-DRA* with Nups occurs in the nucleoplasm. Consistent with previous work [49], this suggests that in both *Drosophila* and human cells there are two pools of nuclear pore proteins: a pool at the NPC and a pool in the nucleoplasm. Although the interaction between genes and Nup100 occurs at the NPC in yeast and the interaction with Nup98 occurs in the nucleoplasm in human cells, the biochemical outputs of these interactions are conserved.

After removing IFN-γ, the *HLA-DRA* gene shows increased colocalization with PML bodies, nuclear “dots” that are enriched for the promyelocytic leukemia factor (PML) [31]. PML bodies increase in number after IFN-γ treatment and depletion of PML led to a decrease in both the rate of *HLA-DRA* reactivation and loss of H3K4me2 after removing IFN-γ [31]. This suggests that relocalization of *HLA-DRA* to these structures is required for IFN-γ memory. Foci of Nup98 in the nucleoplasm do not colocalize with PML bodies [49], suggesting that *HLA-DRA* may not colocalize with Nup98 foci. It will be important to understand how PML bodies and nuclear pore proteins impact each other in this process.

The role of H2A.Z in transcriptional memory is controversial. Whereas H2A.Z is required for *INO1* transcriptional memory and is intimately connected to MRS function [18], loss of H2A.Z affects both the rate of activation and reactivation of *GAL* genes and its role in *GAL* gene transcriptional memory has been challenged [77]–[79]. The MRS from the *INO1* promoter is sufficient to induce both incorporation of H2A.Z and dimethylation of H3K4. However, loss of H2A.Z did not block dimethylation of H3K4, suggesting that H3K4me2 occurs upstream of, or in parallel to, H2A.Z deposition. In HeLa cells, we observed only a slight increase in H2A.Z levels associated with the *HLA-DRA* promoter after removing IFN-γ (Figure S5F). Although a large increase in H2A.Z association is not necessary for H2A.Z to play a role in memory, it is possible that H2A.Z functions as a gene-specific regulator of a more general system to promote reactivation.

### Transcriptional Memory in Animals

Our results suggest that, under certain circumstances, Nup98 regulates H3K4 methylation of a gene that colocalizes with PML bodies. Translocations that result in fusion of PML with the retinoic acid receptor α lead to loss of PML bodies, altered transcription, and acute promyelocytic leukemia [80],[81]. Translocations that result in fusion of Nup98 to transcription factors lead to acute myeloid leukemias [82]–[85]. Finally, translocations that result in fusions of >60 different genes with the H3K4 methyltransferase MLL result in acute lymphoblastic leukemia, in part through altered Hox gene expression [86]–[88]. These striking similarities raise the possibility that these translocations impact the expression of an overlapping set of genes through similar mechanisms, perhaps involving transcriptional priming.

Transcriptional memory plays a broad role in gene priming of IFN-γ-responsive genes (Figure 4). Hundreds of genes displayed faster or stronger induction kinetics in cells that have previously been exposed to IFN-γ and that this effect persists for days in rapidly doubling HeLa cells. This suggests that transcriptional memory can qualitatively alter the response of a system to a particular stimulus. If so, it is possible that the response of cells to other stimuli can be modulated by transcriptional memory. For example, similar to the phenomenon of stress cross-protection in yeast [19], the rate of induction in response to one cytokine could also be qualitatively or quantitatively altered by previous exposure to a different cytokine. Because transcriptional memory is epigenetically inherited through several cell divisions, it could alter the response of cells, tissues, or whole organisms to persistent or episodic stimuli over long timescales. If so, then it might play an important role in pathological inflammation [89],[90].

## Materials and Methods

### Chemicals and Reagents

Unless otherwise noted, chemicals used were obtained from Sigma Aldrich and enzymes were from New England Biolabs. BACs were from Invitrogen. 8WG16 antibody was obtained from Covance, anti-Ser5P CTD (cat no. ab5408), anti-Phe-x-Phe-Gly m414 (cat no. ab50008), anti-Nup98 (cat no. ab45584), anti-Nup96 (cat no. ab124980), anti-Nup107 (cat no. ab85916), anti-H2A.Z (cat no. ab4174), anti-H3K4me2 (cat no. ab32356), and anti-H3K4me3 (cat no. ab1012) were from AbCam. IFN-γ was from PBL Biomedical.

### Yeast Strains

Yeast strains used in this study are listed in Table S6. Strains with the MRS or the *mrs mutant* elements have been described [18] and were created as described [15].

### Chromatin Immunoprecipitation

For yeast experiments, ChIP was performed as described [18]. For TAP-tagged ChIP experiments, Pan Mouse IgG Dynabeads from Invitrogen were used. For HeLa experiments, cells were trypsinized and fixed using 1% formaldehyde for 15 min at 25°C. Cross-linking was quenched using 150 mM glycine, and cells were harvested by centrifugation and washed twice with ice-cold PBS. Cells were lysed in 10 ml MC lysis buffer (10 mM NaCl, 10 mM Tris-HCl, 3 mM MgCl_2_, 0.5% NP-40) and nuclei were recovered by centrifugation at 1,350 rpm twice and snap frozen in liquid nitrogen. Fixed nuclei were resuspended in 1 ml lysis buffer (10 mM Tris-HCl, 100 mM NaCl, 1 mM EDTA, 0.5 mM EGTA, 0.5% N-lauroylsarcosine) with protease inhibitors (Roche) and sonicated using a Branson 450 microtip 16 times for 15 s at setting 5 to generate ∼500 bp average fragment size. 1% Triton X-100 and 0.1% sodium deoxycholate were added back and then chromatin was spun at ∼16,100× *g* at 4°C for 15 min. The supernatant fraction was added to antibody and Dynabeads overnight at 4°C. The beads were recovered and washed four times with lysis buffer+Triton X-100 and sodium deoxycholate. Immunoprecipitated chromatin was eluted in 100 µl TE+1% SDS at 65°C for 15 min. Input and IP fractions were treated with 50 µg RNase A and 100 µg Proteinase K for 1 h at 42°C, before reversing crosslinks overnight at 65°C. DNA was extracted with phenol∶chloroform∶isoamyl alcohol, and chloroform and 2 µg linear acrylamide was added prior to ethanol precipitation. Samples were washed with 70% ethanol and resuspended in 30 µl TE. qPCR was performed as described [12] using oligonucleotides listed in Table S7.

### Cell Culture

For reactivation experiments, HeLa cells were grown to ∼50% confluence, treated with 50 ng/mL of IFN-γ in DMEM supplemented with calf serum and antibiotics for 24 h, washed extensively with PBS, trypsinized, and seeded to plates at appropriate densities that would lead to the same confluence when the cells were harvested. Transfections were performed using Lipofectamine 2000 (Invitrogen) and siRNA smart pools for Nup98, Nup107, or scrambled (Thermo Fisher) according to the manufacturer's recommendations. RNA was harvested using Trizol Reagent (Invitrogen) according to the manufacturer's recommendations.

### DNA-FISH

HeLa cells were treated with IFN-γ as indicated. Cells were then trypsinized and adhered to polylysine-treated slides. Cells were fixed with formaldehyde for 15 min at 25°C and then washed with PBS+0.5% Triton X-100 several times. Slides were then treated with 0.1 M HCl on ice for 15 min, and then in 50% formamide/2× SSC for 30 min at 80°C. Fish probes were generated from BACs using FISH Tag DNA kit 488 (Invitrogen). Probes were added to coverslip and then cells were covered, sealed with rubber cement, and heated at 80°C for 4 min, followed by incubation overnight at 37°C in the dark. Slides were then washed 3 times with 2× SSC at 37°C, 3 times with 0.1× SSC, and stained with Hoechst in 0.1× SSC at 25°C for 10 min, followed by 4× SSC/0.2% Tween-20, mounted in Vectashield, sealed with nail polish, and *z* stacks of images were obtained using a Leica SP5 confocal microscope with a 100× objective. Measurements of the distance from FISH probe signal to nuclear periphery were made using ImageJ for individual *z* slices.

### RT qPCR and Microarrays

HeLa cells were treated with IFN-γ for 0, 6, or 24 h for initial activation or after a previous 24 h treatment followed by a 48 h rest period (reactivation). RNA samples were isolated using Trizol (Invitrogen). RNA was DNase I treated and then reverse transcribed using Superscript III (Invitrogen). For qPCR experiments, primer locations are shown in Figure S3. For microarrays, the second strand was synthesized using second strand synthesis kit (New England Biolabs). cDNA from two biological replicates was then labeled and hybridized to Agilent 128×135K arrays using human genome build hg18. Log_2_ ratios were generated using DNAstar Arraystar software (Roche). Averaged, normalized array data for the subset of genes that were induced ≥2 fold on average between 6 h and 24 h were organized using *k* means clustering by Cluster and visualized using Treeview.

### Western Blotting

Media was removed from cells, and cells were scrapped off of plates in PBS using a rubber scraper. Cells were pelleted at 1,500 rpm at 4°C. Pellets were resuspended in whole cell extract buffer (50 mM Tris, 280 mM NaCl, 0.5% NP-40, 0.2 mM EDTA, 2 mM EGTA, 10% glycerol) with DTT, sodium vanadate, and protease inhibitors. Lysates were incubated on ice for 20 min and then spun at 13,200 rpm at 4°C. Supernatant was harvested, and protein concentration was quantified using BCA assay (Pierce) and frozen in liquid nitrogen. 75 µg of each sample was separated on a 10% SDS Tris-MOPS gel, transferred to nitrocellulose, and incubated overnight with antibodies against Nup98 GAPDH in TBST+5% skim milk at 4°C. Blots were then washed twice with TBST, incubated with secondary antibody conjugated to HRP, and exposed to Enhanced Chemiluminescence reagents (Pierce) and imaged using a UVP BiospectrumAC Imaging System.

### Chromatin Localization Assay

Yeast strains were harvested, fixed with methanol, and processed for microscopy as described [91].

### HeLa Cell Immunofluorescence

Cells were harvested by centrifugation, washed 3 times with PBS, and adhered to polylysine-treated slides for 3 min at RT. Cells were fixed with 4% paraformaldehyde in PBS at RT for 15 min, washed twice with PBS, and then permeabilized with PBS+0.5% Triton X-100 at RT for 30 min. Cells were then blocked with PBS+3% BSA+0.1% Triton X-100 for 1 h at room temperature, and then incubated with primary antibodies (either m414 with Nup96 or m414 with Nup98) overnight at 4°C. The following day, cells were washed twice with PBS and then incubated with secondary antibody (Goat anti Rabbit Alexafluor488 or Goat anti Mouse Alexafluor 594) in blocking buffer for 2 h at room temperature, washed twice with PBS, and mounted with Vectashield. Cells were imaged on a Leica SP5 confocal microscope.

## Supporting Information

Figure S1 Phospho-Ser5 RNAPII associates with the active but not the recently repressed *INO1* promoter. Wild-type and *mrs mutant* cells were grown in repressing (+inositol, black bars), activating (−inositol, dark grey bars), or recently repressed (−ino→+ino 3 h light grey bars) conditions and ChIP was performed. Recovery of the *INO1* promoter and the repressed *GAL1* promoter was quantified relative to input by qPCR. Error bars represent the standard error of the mean for three experiments.
(TIF)

Figure S2 *HLA-DR*A induction in response to IFN-γ. (A) Cells were washed and split at t = 0 h. Cells were harvested at the indicated times and *HLA-DRA* mRNA levels were quantified relative to *β*-*ACTIN* by RT-qPCR. (B) Cells were treated with IFN-γ for either 2 h or 24 h. Cells treated for 2 h were either harvested immediately or harvested 24 h after removing IFN-γ, either after washing or after splitting. Alternatively, cells were treated after trypsinizing for 2 h. *HLA-DRA* mRNA was quantified relative to *β*-*ACTIN* by RT-qPCR. (C) Rate of activation and reactivation of *HLA-DRA*. For reactivation experiments, cells were first treated for either 24 h or 2 h before splitting and allowing the cells to grow for 48 h.
(TIF)

Figure S3 Location of human qPCR primer pairs. The positions of PCR products generated by qPCR primers are mapped against the human genome. Exons are represented by thick bars, untranslated regions are represented by medium bars, and introns are represented by thin bars. Scale bars are gene-specific.
(TIF)

Figure S4 The position of the *CIITA* locus with respect to the nuclear periphery before, during, and after IFN-γ treatment. DNA-FISH was performed using cells treated as indicated in Figure 2A. Measurements from foci to edge of Hoescht staining for ≥200 foci were binned, and the distribution within the population was plotted for each condition. Black, uninduced cells; blue, 24 h of induction with IFN-γ; red, 48 h after the removal of IFN-γ. Distances were binned into 0.4 µm bins and the distribution of distances within the population was blotted. Mean distances to the nuclear periphery and standard deviations for each mean are shown for each distribution.
(TIF)

Figure S5 Genes exhibiting transcriptional memory. (A–D) Expression of *CIITA* (A), *OAS2* (B), *HLA-DPB1* (C), and *HLA-DQB1* (D) during activation and reactivation. RT qPCR expression data for each of the candidate genes quantified relative to *β*-*ACTIN*. Cells were harvested at the indicated times during activation or reactivation. For reactivation experiments, cells were split after treatment with IFN-γ and then allowed to grow for 48 h before adding IFN-γ. Black squares, activation; grey squares, reactivation. (E and F) Cells were treated as schematized in Figure 2B, and ChIP was performed using anti-RNAPII (E) or H2A.Z (F), and both promoter and coding sequences for each gene were quantified relative to input by qPCR. Black, uninduced; dark grey, treatment with IFN-γ for 24 h; light grey, treatment with IFN-γ for 24 h 48 h after removal of IFN-γ; white, 96 h after removal of IFN-γ. For all panels, error bars represent the standard error of the mean for three experiments.
(TIF)

Figure S6 H3K4 methylation in transcriptional memory. (A and B) Cells were treated as schematized in Figure 2B, and ChIP was performed against H3K4me3 (A) and H3K4me2 (B). Promoter and coding sequence were quantified using qPCR for indicated genes. Black, uninduced; dark grey, treatment with IFN-γ for 24 h; light grey, treatment with IFN-γ for 24 h 48 h after removal of IFN-γ; white, 96 h postremoval of IFN-γ. (C and D) Yeast cells were grown under repressing (+inositol, black bars), activating (−inositol, dark grey), and recently repressed (−ino→+ino, light grey) conditions, fixed and processed for ChIP against H3K4me3. For panel C, strains having either the MRS or the nonfunctional *mrs mutant* inserted at *URA3* were grown, and recovery of the *INO1* promoter, the insertion site at *URA3*, and the coding sequence of the repressed *PRM1* gene was quantified by qPCR relative to input. For panel D, wild-type and *htz1*Δ strains were grown, and recovery of both the *INO1* promoter and the *PRM1* coding sequence was quantified by qPCR relative to input. For all panels, error bars represent the standard error of the mean for three experiments.
(TIF)

Figure S7 Immunofluorescence against m414 and Nup98 in cells treated with siRNAs. Cells treated with either scrambled or *NUP98* siRNAs were fixed and processed for immunofluorescence against m414 or Nup98.
(TIF)

Figure S8 ChIP against H3K4me3 in wild-type and *nup100*Δ cells. Wild-type and *nup100*Δ cells grown in repressing (+inositol, black), activating (−inositol, dark grey), and recently repressed conditions (−ino→+ino 3 h, light grey) were subjected to ChIP against H3K4me3. Recovery of the *INO1* promoter and the *PRM1* coding sequence were quantified by qPCR. Error bars represent the standard error of the mean for three experiments.
(TIF)

Figure S9 Immunoblot against Nup96 in siRNA-treated cells. Protein extracts from cells treated with either scrambled or *NUP98* siRNAs were separated by SDS PAGE and probed using antibodies against either Nup96 or GAPDH.
(TIF)

Table S1 IFN-γ-induced genes organized into clusters. For Tables S2–S5, 24,251 genes were used as the background set. Of these, 16,766 were associated with Gene Ontology terms (GO terms; 08/04/12 GO database).
(XLSX)

Table S2 Top gene ontology terms enriched among IFN-γ-induced genes. For Table S2, the set of genes associated with GO terms was compared with the 544 genes that were ≥2.0-fold induced in response to IFN-γ. Listed are the number of genes having that GO term and the number of IFN-γ-induced genes having that GO term. *p* values were calculated by GOrilla using HG or mHG models [50]. FDR q values are corrected using the method of [92].
(DOCX)

Table S3 Top gene ontology terms enriched among cluster 1 genes. For Table S3, the 16,766 genes associated with GO terms were compared with the 189 genes in cluster 1 that were associated with GO terms. Listed are the number of genes having that GO term and the number of genes in the cluster having that GO term.
(DOCX)

Table S4 Top gene ontology terms enriched among cluster 2 genes. For Table S4, the 16,766 genes associated with GO terms were compared with the 315 genes in cluster 2 that were associated with GO terms. Listed are the number of genes having that GO term and the number of genes in the cluster having that GO term.
(DOCX)

Table S5 Top gene ontology terms enriched among cluster 3 genes. For Table S5, the 16,766 genes associated with GO terms were compared with the 40 genes in cluster 3 that were associated with GO terms. Listed are the number of genes having that GO term and the number of genes in the cluster having that GO term.
(DOCX)

Table S6 Yeast strains used in this study.
(DOCX)

Table S7 Oligonucleotides used in this study.
(DOCX)

## Abbreviations

BACs: bacterial artificial chromosomes
ChIP: chromatin immunoprecipitation
CTD: carboxy terminal domain
DSIF: DRB sensitivity-inducing factor
FISH: fluorescence in situ hybridization
GO: gene ontology
GRS: gene recruitment sequence
H3K4me: histone H3 lysine 4 methylation
IFN-γ: interferon gamma
MRS: memory recruitment sequence
NELF: negative elongation factor
NPC: nuclear pore complex
Nups: nuclear pore proteins
PIC: preinitiation complex
PML: promyelocytic leukemia factor
RNAPII: RNA polymerase II
TAP: tandem affinity purification
