# Supplementary figures and images for: A Conserved Role for Human Nup98 in Altering Chromatin Structure and Promoting Epigenetic Transcriptional Memory
Source: PLoS Biol. 2013 Mar 26;11(3):e1001524. doi: 10.1371/journal.pbio.1001524 (PMC3608542; doi:10.1371/journal.pbio.1001524)

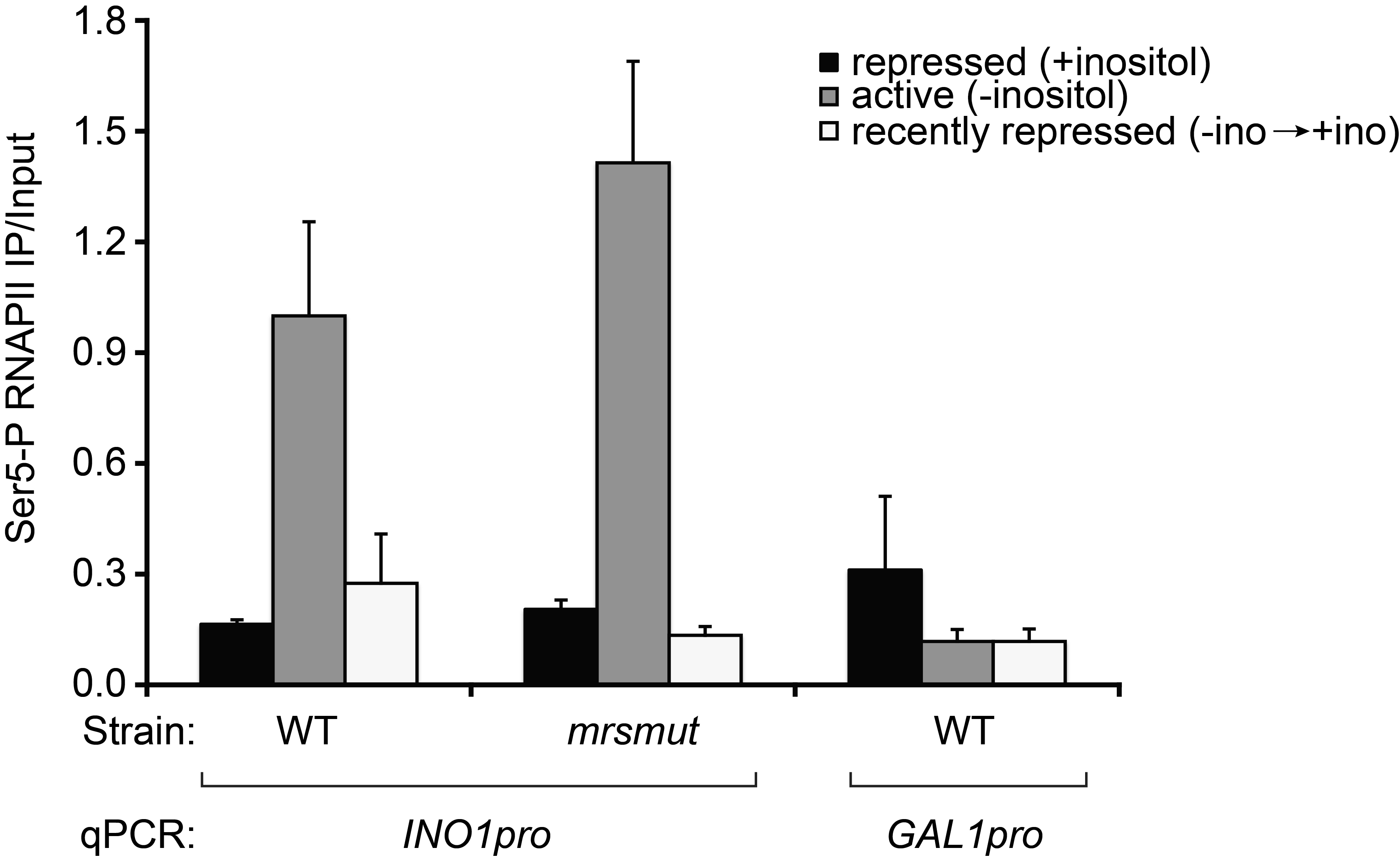

Supplement: Figure S1 — Phospho-Ser5 RNAPII associates with the active but not the recently repressed INO1 promoter. Wild-type and mrs mutant cells were grown in repressing (+inositol, black bars), activating (−inositol, dark grey bars), or recently repressed (−ino→+ino 3 h light grey bars) conditions and ChIP was performed. Recovery of the INO1 promoter and the repressed GAL1 promoter was quantified relative to input by qPCR. Error bars represent the standard error of the mean for three experiments. (TIF) [file pbio.1001524.s001.tif]

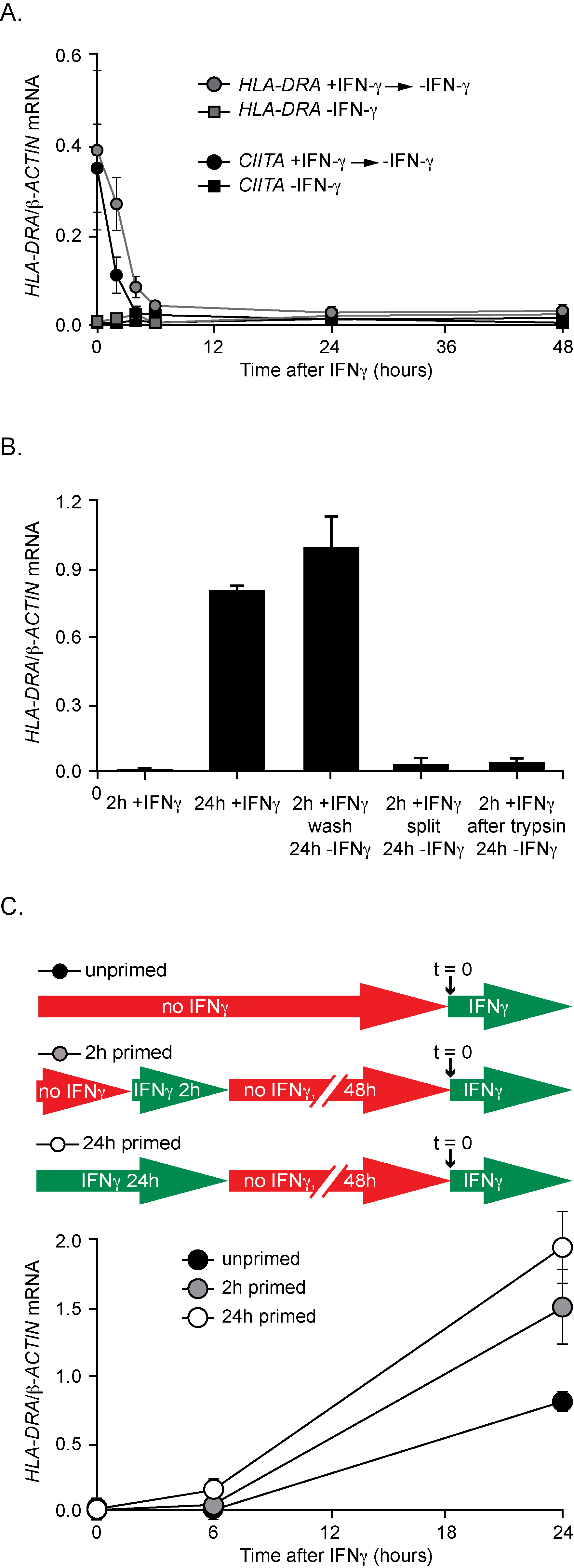

Supplement: Figure S2 — HLA-DRA induction in response to IFN-γ. (A) Cells were washed and split at t = 0 h. Cells were harvested at the indicated times and HLA-DRA mRNA levels were quantified relative to β-ACTIN by RT-qPCR. (B) Cells were treated with IFN-γ for either 2 h or 24 h. Cells treated for 2 h were either harvested immediately or harvested 24 h after removing IFN-γ, either after washing or after splitting. Alternatively, cells were treated after trypsinizing for 2 h. HLA-DRA mRNA was quantified relative to β-ACTIN by RT-qPCR. (C) Rate of activation and reactivation of HLA-DRA. For reactivation experiments, cells were first treated for either 24 h or 2 h before splitting and allowing the cells to grow for 48 h. (TIF) [file pbio.1001524.s002.tif]

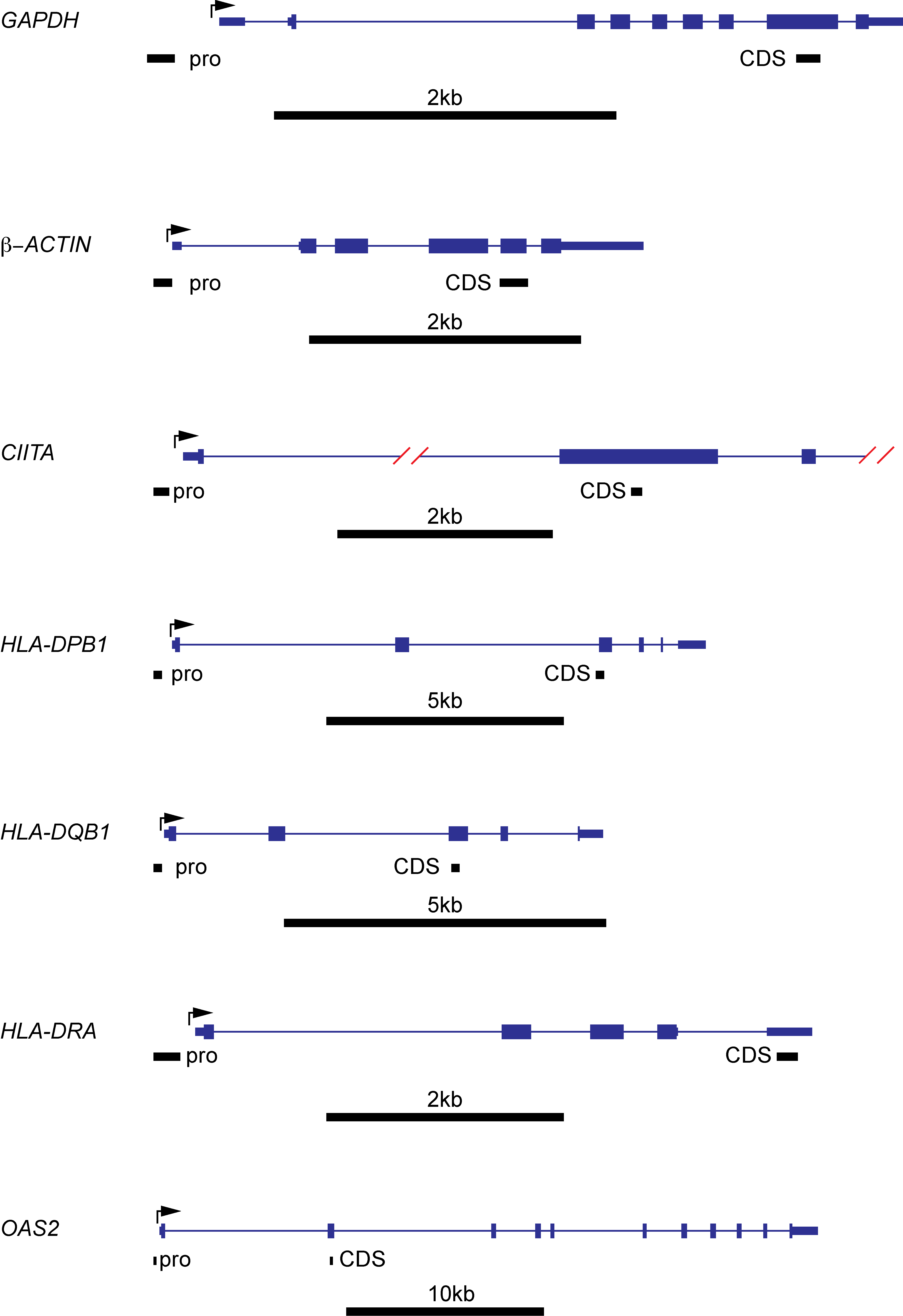

Supplement: Figure S3 — Location of human qPCR primer pairs. The positions of PCR products generated by qPCR primers are mapped against the human genome. Exons are represented by thick bars, untranslated regions are represented by medium bars, and introns are represented by thin bars. Scale bars are gene-specific. (TIF) [file pbio.1001524.s003.tif]

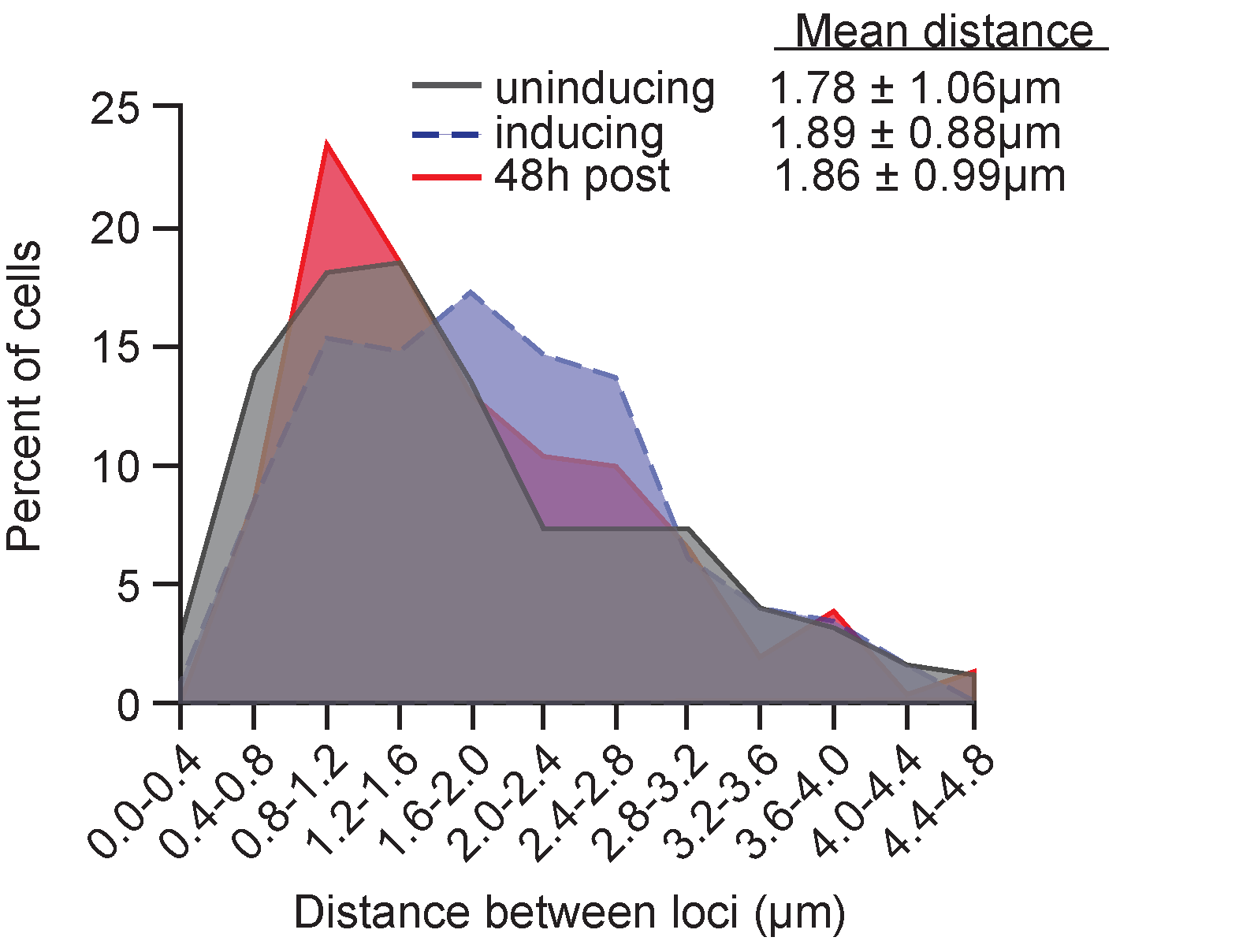

Supplement: Figure S4 — The position of the CIITA locus with respect to the nuclear periphery before, during, and after IFN-γ treatment. DNA-FISH was performed using cells treated as indicated in Figure 2A. Measurements from foci to edge of Hoescht staining for ≥200 foci were binned, and the distribution within the population was plotted for each condition. Black, uninduced cells; blue, 24 h of induction with IFN-γ; red, 48 h after the removal of IFN-γ. Distances were binned into 0.4 µm bins and the distribution of distances within the population was blotted. Mean distances to the nuclear periphery and standard deviations for each mean are shown for each distribution. (TIF) [file pbio.1001524.s004.tif]

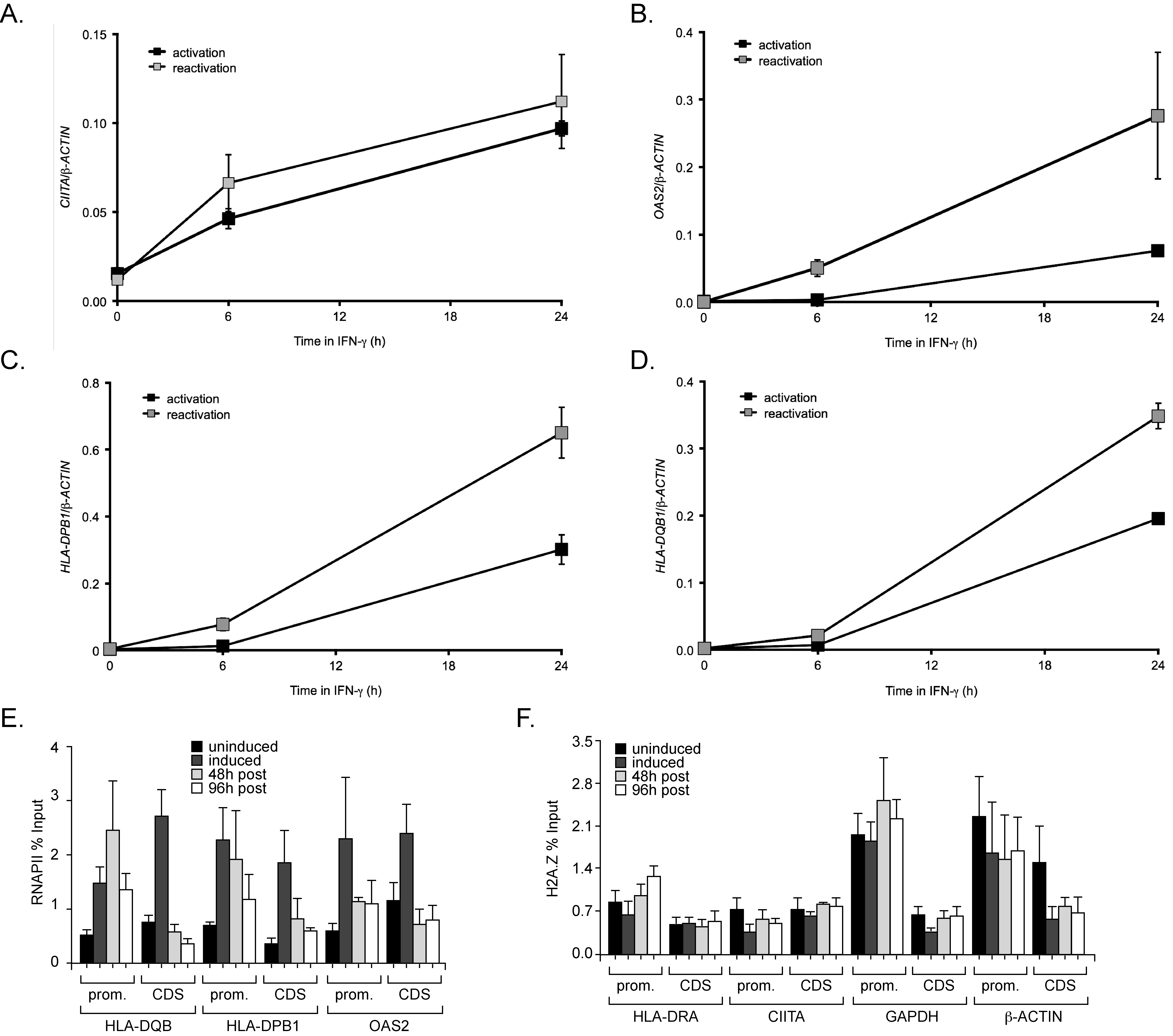

Supplement: Figure S5 — Genes exhibiting transcriptional memory. (A–D) Expression of CIITA (A), OAS2 (B), HLA-DPB1 (C), and HLA-DQB1 (D) during activation and reactivation. RT qPCR expression data for each of the candidate genes quantified relative to β-ACTIN. Cells were harvested at the indicated times during activation or reactivation. For reactivation experiments, cells were split after treatment with IFN-γ and then allowed to grow for 48 h before adding IFN-γ. Black squares, activation; grey squares, reactivation. (E and F) Cells were treated as schematized in Figure 2B, and ChIP was performed using anti-RNAPII (E) or H2A.Z (F), and both promoter and coding sequences for each gene were quantified relative to input by qPCR. Black, uninduced; dark grey, treatment with IFN-γ for 24 h; light grey, treatment with IFN-γ for 24 h 48 h after removal of IFN-γ; white, 96 h after removal of IFN-γ. For all panels, error bars represent the standard error of the mean for three experiments. (TIF) [file pbio.1001524.s005.tif]

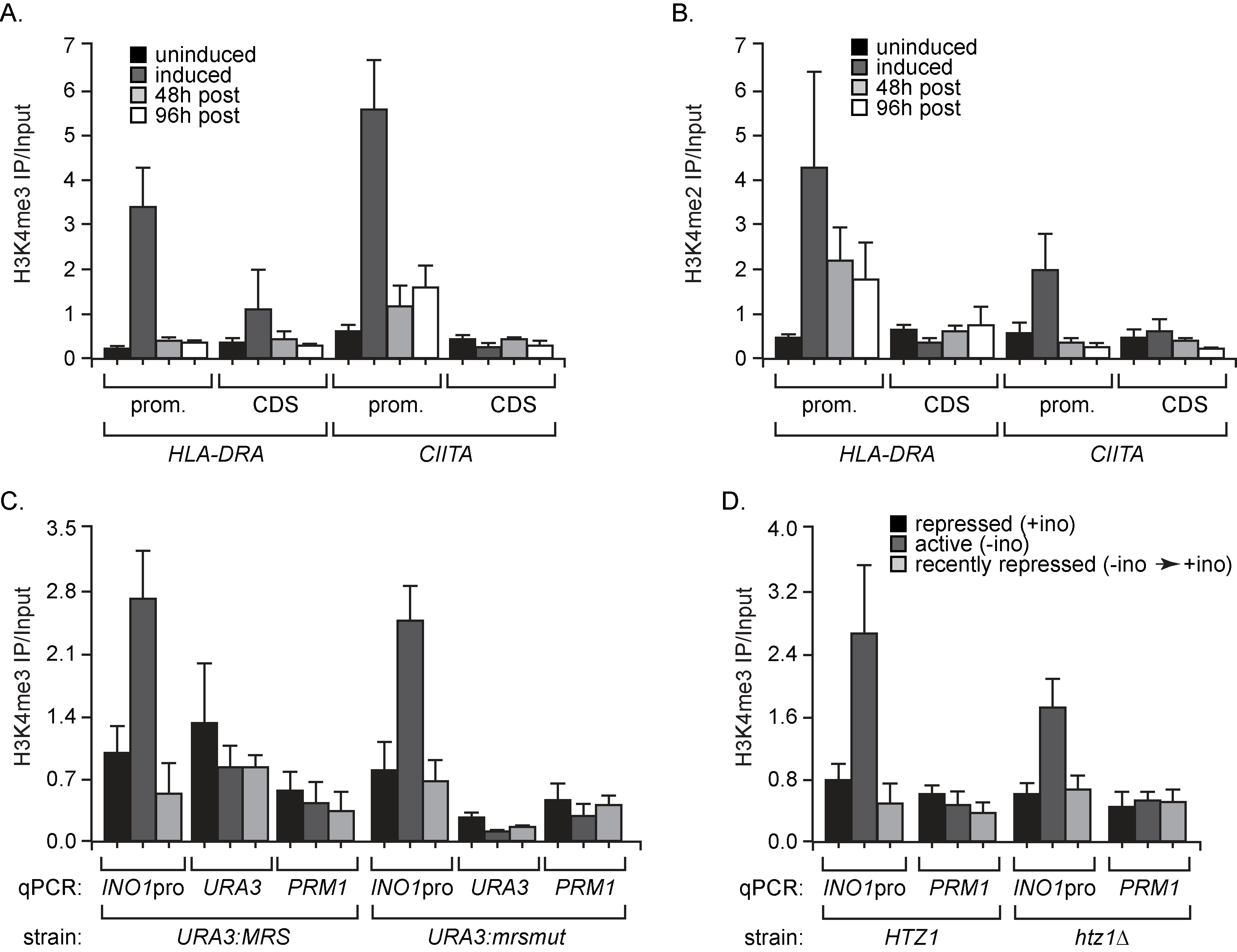

Supplement: Figure S6 — H3K4 methylation in transcriptional memory. (A and B) Cells were treated as schematized in Figure 2B, and ChIP was performed against H3K4me3 (A) and H3K4me2 (B). Promoter and coding sequence were quantified using qPCR for indicated genes. Black, uninduced; dark grey, treatment with IFN-γ for 24 h; light grey, treatment with IFN-γ for 24 h 48 h after removal of IFN-γ; white, 96 h postremoval of IFN-γ. (C and D) Yeast cells were grown under repressing (+inositol, black bars), activating (−inositol, dark grey), and recently repressed (−ino→+ino, light grey) conditions, fixed and processed for ChIP against H3K4me3. For panel C, strains having either the MRS or the nonfunctional mrs mutant inserted at URA3 were grown, and recovery of the INO1 promoter, the insertion site at URA3, and the coding sequence of the repressed PRM1 gene was quantified by qPCR relative to input. For panel D, wild-type and htz1Δ strains were grown, and recovery of both the INO1 promoter and the PRM1 coding sequence was quantified by qPCR relative to input. For all panels, error bars represent the standard error of the mean for three experiments. (TIF) [file pbio.1001524.s006.tif]

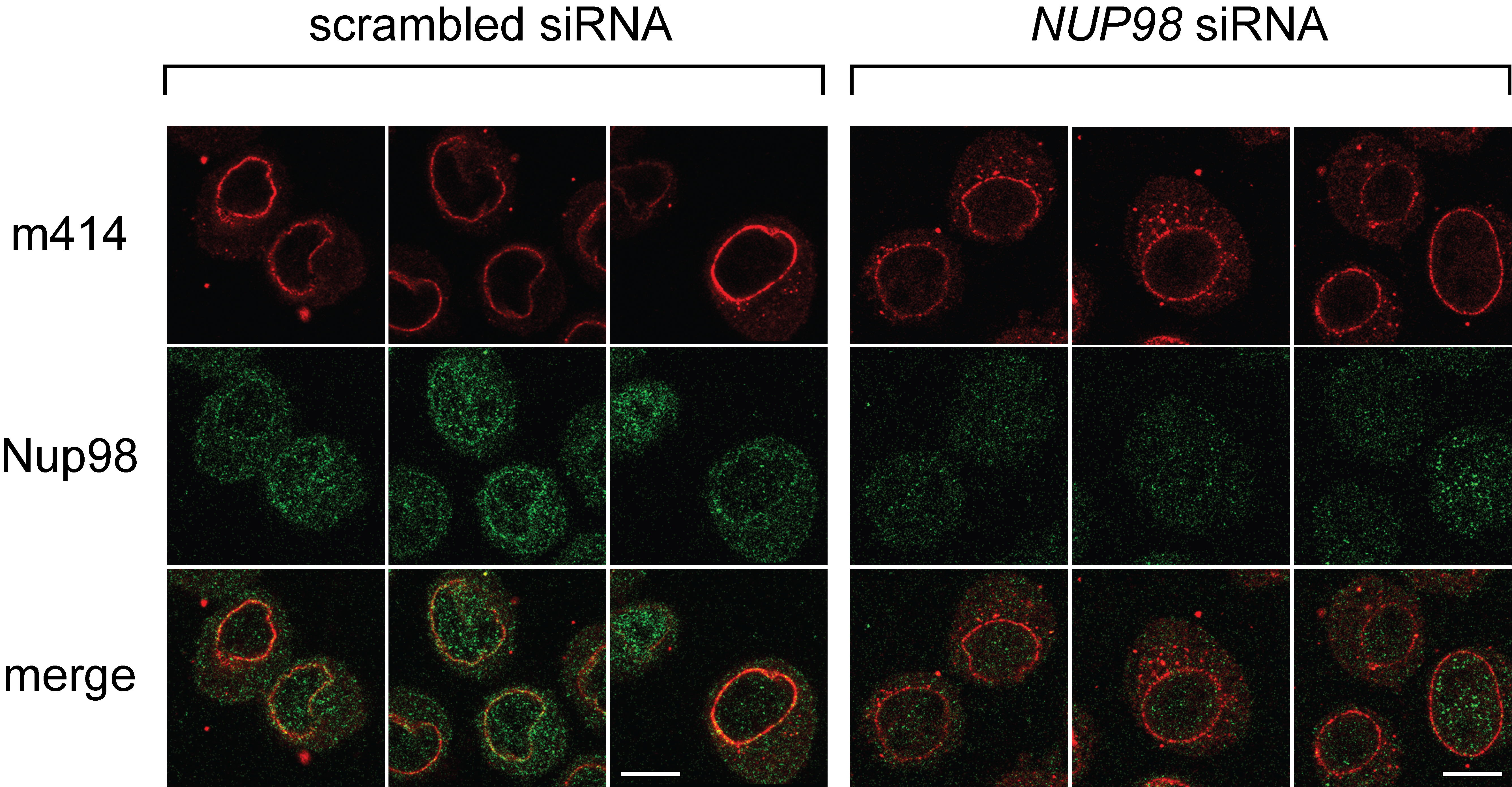

Supplement: Figure S7 — Immunofluorescence against m414 and Nup98 in cells treated with siRNAs. Cells treated with either scrambled or NUP98 siRNAs were fixed and processed for immunofluorescence against m414 or Nup98. (TIF) [file pbio.1001524.s007.tif]

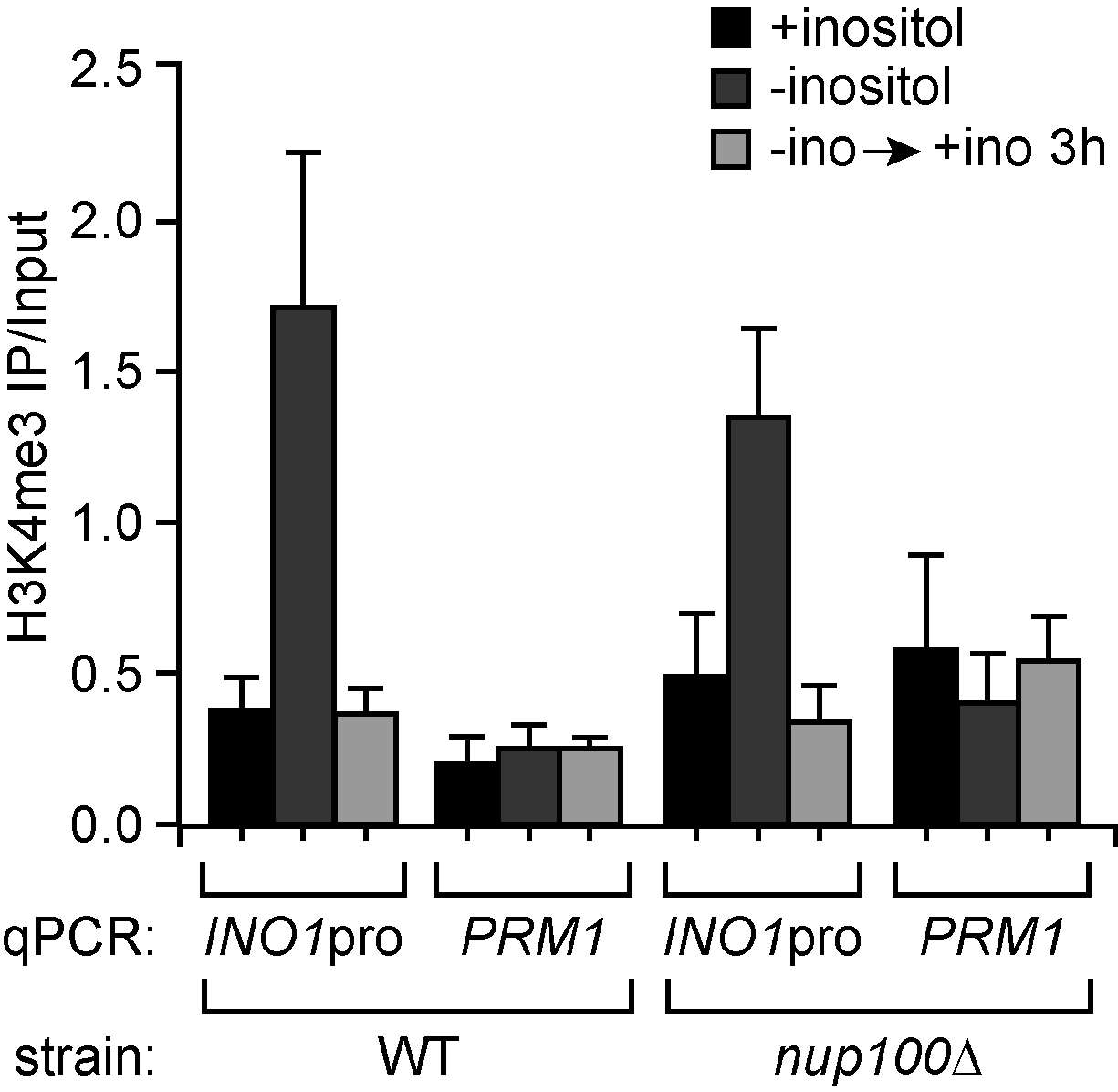

Supplement: Figure S8 — ChIP against H3K4me3 in wild-type and nup100Δ cells. Wild-type and nup100Δ cells grown in repressing (+inositol, black), activating (−inositol, dark grey), and recently repressed conditions (−ino→+ino 3 h, light grey) were subjected to ChIP against H3K4me3. Recovery of the INO1 promoter and the PRM1 coding sequence were quantified by qPCR. Error bars represent the standard error of the mean for three experiments. (TIF) [file pbio.1001524.s008.tif]

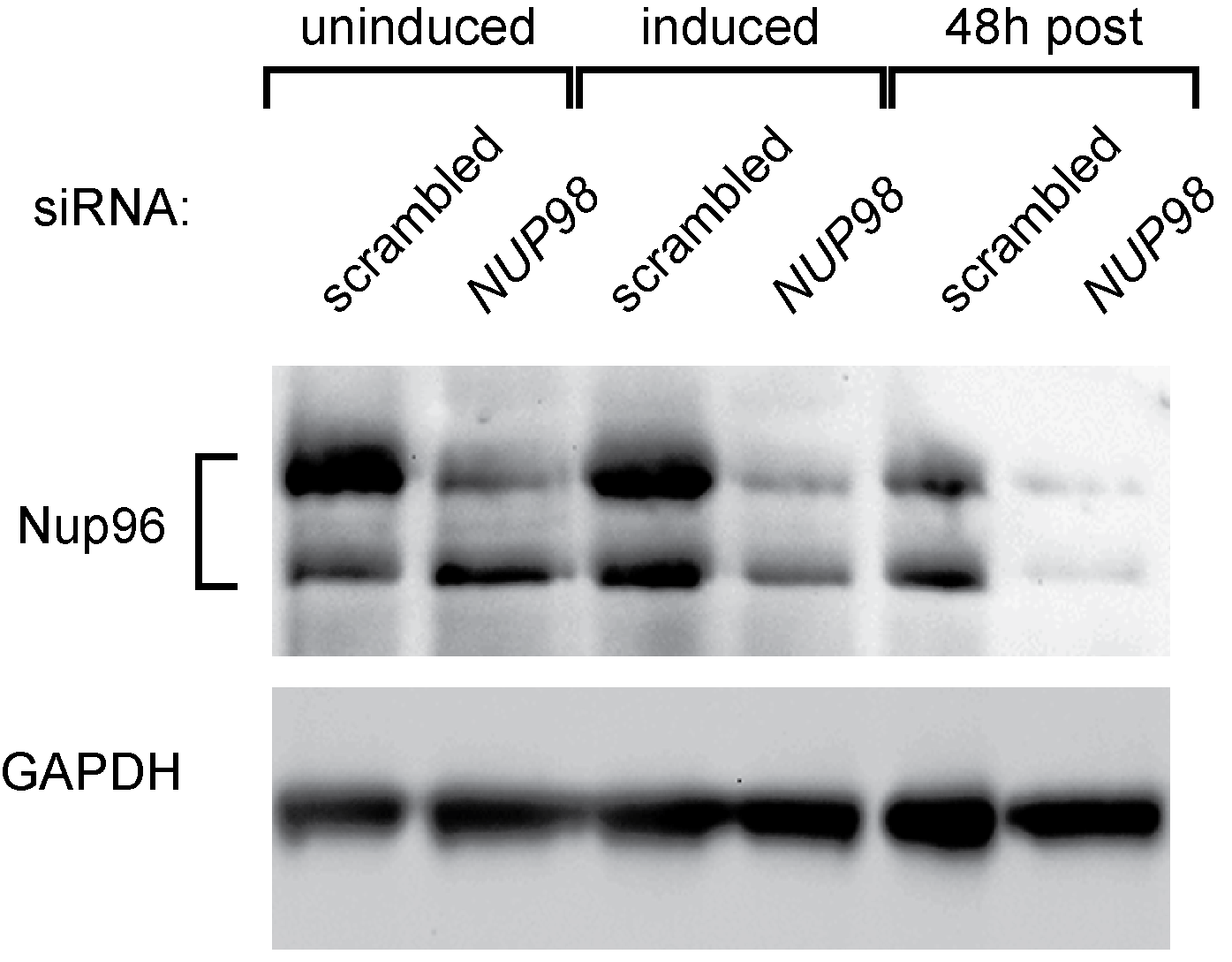

Supplement: Figure S9 — Immunoblot against Nup96 in siRNA-treated cells. Protein extracts from cells treated with either scrambled or NUP98 siRNAs were separated by SDS PAGE and probed using antibodies against either Nup96 or GAPDH. (TIF) [file pbio.1001524.s009.tif]
