# Supplementary material for: A Conserved Role for Human Nup98 in Altering Chromatin Structure and Promoting Epigenetic Transcriptional Memory
Source: PLoS Biol. 2013 Mar 26;11(3):e1001524. doi: 10.1371/journal.pbio.1001524 (PMC3608542; doi:10.1371/journal.pbio.1001524)
Supplement: Table S2 — Top gene ontology terms enriched among IFN-γ-induced genes. For Table S2, the set of genes associated with GO terms was compared with the 544 genes that were ≥2.0-fold induced in response to IFN-γ. Listed are the number of genes having that GO term and the number of IFN-γ-induced genes having that GO term. p values were calculated by GOrilla using HG or mHG models [50]. FDR q values are corrected using the method of [92]. (DOCX) [file pbio.1001524.s011.docx]

**Table S2. Top gene ontology terms enriched among IFN-γ-induced genes**

| GO term | Description | *P* | FDR q | Number in GO | Number in both |
| --- | --- | --- | --- | --- | --- |
| 0002682 | Regulation of immune system process | 7.76 x 10^-23^ | 8.45 x 10^-19^ | 811 | 86 |
| 0006955 | Immune response | 3.20 x 10^-20^ | 1.74 x 10^-16^ | 683 | 74 |
| 0002376 | Immune system process | 4.74 x 10^-19^ | 1.72 x 10^-15^ | 1170 | 99 |
| 0050776 | Regulation of immune response | 8.06 x 10^-19^ | 2.20 x 10^-15^ | 496 | 60 |
| 0009607 | Response to biotic stimulus | 1.86 x 10^-18^ | 4.05 x 10^-15^ | 520 | 61 |
| 0034341 | Response to interferon-gamma | 3.99 x 10^-18^ | 7.24 x 10^-15^ | 98 | 27 |
| 0034097 | Response to cytokine stimulus | 1.16 x 10^-17^ | 1.81 x 10^-14^ | 462 | 56 |
| 0002684 | Positive regulation of immune system process | 1.45 x 10^-17^ | 1.98 x 10^-14^ | 495 | 58 |
| 0071346 | Cellular response to interferon-gamma | 6.34 x 10^-17^ | 7.67 x 10^-14^ | 82 | 24 |
| 0071345 | Cellular response to cytokine stimulus | 2.12 x 10^-16^ | 2.30 x 10^-13^ | 371 | 48 |
| 0019221 | Cytokine-mediated signaling pathway | 6.40 x 10^-16^ | 6.34 x 10^-13^ | 296 | 42 |
| 0048583 | Regulation of response to stimulus | 8.03 x 10^-16^ | 7.29 x 10^-13^ | 2043 | 133 |
| 0060333 | Interferon-gamma signaling pathway | 1.20 x 10^-15^ | 1.00 x 10^-12^ | 67 | 21 |
| 0002504 | Antigen processing and presentation of peptide or polysaccharide antigen via MHC class II | 1.88 x 10^-14^ | 1.46 x 10^-11^ | 18 | 12 |
| 0050896 | Response to stimulus | 5.71 x 10^-14^ | 4.15 x 10^-11^ | 5581 | 264 |
| 0010033 | Response to organic substance | 9.27 x 10^-14^ | 6.13 x 10^-11^ | 1602 | 108 |
| 0031347 | Regulation of defense response | 1.09 x 10^-13^ | 6.96 x 10^-11^ | 388 | 45 |
| 0051707 | Response to other organism | 1.30 x 10^-13^ | 7.86 x 10^-11^ | 344 | 42 |
| 0006952 | Defense response | 3.00 x 10^-13^ | 1.72 x 10^-10^ | 783 | 67 |
| 0070887 | Cellular response to chemical stimulus | 3.75 x 10^-13^ | 2.04 x 10^-10^ | 1247 | 90 |
| 0009615 | Response to virus | 4.11 x 10^-13^ | 2.13 x 10^-10^ | 200 | 31 |

Notes: for tables S2-S5, 24,251 genes were used as the background set. Of these, 16,766 were associated with Gene Ontology terms (GO terms; 08/04/12 GO database).

For Table S2 the set of genes associated with GO terms was compared with the 544 genes that were ≥ 2.0 fold induced in response to IFN-γ. Listed are the number of genes having that GO term and the number of IFN-γ-induced genes having that GO term.

*P* values were calculated by GOrilla using HG or mHG models [[1](#_ENREF_1)]

FDR q values are corrected using the method of [[2](#_ENREF_2)]

**Supplementary References**

1. Eden E, Navon R, Steinfeld I, Lipson D, Yakhini Z (2009) GOrilla: a tool for discovery and visualization of enriched GO terms in ranked gene lists. BMC Bioinformatics 10: 48.

2. Hochberg Y, Benjamini Y (1990) More powerful procedures for multiple significance testing. Stat Med 9: 811-818.
