# Supplementary material for: A Conserved Role for Human Nup98 in Altering Chromatin Structure and Promoting Epigenetic Transcriptional Memory
Source: PLoS Biol. 2013 Mar 26;11(3):e1001524. doi: 10.1371/journal.pbio.1001524 (PMC3608542; doi:10.1371/journal.pbio.1001524)
Supplement: Table S3 — Top gene ontology terms enriched among cluster 1 genes. For Table S3, the 16,766 genes associated with GO terms were compared with the 189 genes in cluster 1 that were associated with GO terms. Listed are the number of genes having that GO term and the number of genes in the cluster having that GO term. (DOCX) [file pbio.1001524.s012.docx]

**Table S3. Top gene ontology terms enriched among Cluster 1 genes**

| GO term | Description | *P* | FDR q | Number in GO | Number in both |
| --- | --- | --- | --- | --- | --- |
| 0071346 | Cellular response to interferon-gamma | 7.09 x 10^-12^ | 7.72 x 10^-8^ | 82 | 13 |
| 0034341 | Response to interferon-gamma | 7.30 x 10^-11^ | 3.98 x 10^-7^ | 98 | 13 |
| 0060333 | Interferon-gamma-mediated signaling pathway | 2.07 x 10^-10^ | 7.52 x 10^-7^ | 67 | 11 |
| 0050776 | Regulation of immune response | 4.49 x 10^-8^ | 1.22 x 10^-4^ | 496 | 22 |
| 0050727 | Regulation of inflammatory response | 7.65 x 10^-8^ | 1.67 x 10^-4^ | 172 | 13 |
| 0002252 | Immune effector process | 8.51 x 10^-8^ | 1.55 x 10^-4^ | 205 | 14 |
| 0031347 | Regulation of defense response | 8.58 x 10^-8^ | 1.34 x 10^-4^ | 388 | 19 |
| 0002682 | Regulation of immune system process | 1.22 x 10^-7^ | 1.66 x 10^-4^ | 811 | 28 |
| 0002684 | Positive regulation of immune system process | 1.96 x 10^-7^ | 2.37 x 10^-4^ | 495 | 21 |
| 0006955 | Immune response | 7.82 x 10^-7^ | 8.52 x 10^-4^ | 683 | 24 |
| 0002376 | Immune system process | 8.70 x 10^-7^ | 8.62 x 10^-4^ | 1170 | 33 |
| 0071345 | Cellular response to cytokine stimulus | 1.07 x 10^-6^ | 9.69 x 10^-4^ | 371 | 17 |
| 0071345 | Response to cytokine stimulus | 1.23 x 10^-6^ | 1.03 x 10^-4^ | 462 | 19 |
| 0009607 | Response to biotic stimulus | 1.79 x 10^-6^ | 1.39 x 10^-3^ | 520 | 20 |
| 0050778 | Positive regulation of immune response | 2.42 x 10^-6^ | 1.76 x 10^-3^ | 310 | 15 |
| 0048583 | Regulation of response to stimulus | 2.68 x 10^-6^ | 1.82 x 10^-3^ | 2043 | 46 |
| 0010033 | Response to organic substance | 2.99 x 10^-6^ | 1.91 x 10^-3^ | 1602 | 39 |

For Table S3, the 16,766 genes associated with GO terms were compared with the 189 genes in cluster 1 that were associated with GO terms. Listed are the number of genes having that GO term and the number of genes in the cluster having that GO term.
