# Supplementary material for: A Conserved Role for Human Nup98 in Altering Chromatin Structure and Promoting Epigenetic Transcriptional Memory
Source: PLoS Biol. 2013 Mar 26;11(3):e1001524. doi: 10.1371/journal.pbio.1001524 (PMC3608542; doi:10.1371/journal.pbio.1001524)
Supplement: Table S4 — Top gene ontology terms enriched among cluster 2 genes. For Table S4, the 16,766 genes associated with GO terms were compared with the 315 genes in cluster 2 that were associated with GO terms. Listed are the number of genes having that GO term and the number of genes in the cluster having that GO term. (DOCX) [file pbio.1001524.s013.docx]

**Table S4. Top gene ontology terms enriched among Cluster 2 genes**

| GO term | Description | *P* | FDR q | Number in GO | Number in both |
| --- | --- | --- | --- | --- | --- |
| 0002682 | Regulation of immune system process | 6.62 x 10^-10^ | 7.20 x 10^-6^ | 808 | 43 |
| 0009607 | Response to biotic stimulus | 4.73 x 10^-8^ | 2.57 x 10^-4^ | 517 | 30 |
| 0048583 | Regulation of response to stimulus | 1.41 x 10^-7^ | 5.11 x 10^-4^ | 2036 | 71 |
| 0006955 | Immune response | 6.94 x 10^-7^ | 1.89 x 10^-3^ | 683 | 33 |
| 0050776 | Regulation of immune response | 7.73 x 10^-7^ | 1.68 x 10^-3^ | 495 | 27 |
| 0050896 | Response to stimulus | 1.40 x 10^-6^ | 2.54 x 10^-3^ | 5575 | 145 |
| 0002237 | Response to molecule of bacterial origin | 2.22 x 10^-6^ | 3.45 x 10^-3^ | 209 | 16 |
| 0002376 | Immune system process | 3.41 x 10^-6^ | 4.64 x 10^-3^ | 1169 | 45 |
| 0031347 | Regulation of defense response | 4.18 x 10^-6^ | 5.04 x 10^-3^ | 386 | 22 |
| 0050789 | Regulation of biological process | 7.25 x 10^-6^ | 7.89 x 10^-3^ | 7738 | 184 |
| 0002684 | Positive regulation of immune system process | 7.51 x 10^-6^ | 7.42 x 10^-3^ | 494 | 25 |

For Table S4 the 16,766 genes associated with GO terms were compared with the 315 genes in cluster 2 that were associated with GO terms. Listed are the number of genes having that GO term and the number of genes in the cluster having that GO term.
