# Supplementary material for: A Conserved Role for Human Nup98 in Altering Chromatin Structure and Promoting Epigenetic Transcriptional Memory
Source: PLoS Biol. 2013 Mar 26;11(3):e1001524. doi: 10.1371/journal.pbio.1001524 (PMC3608542; doi:10.1371/journal.pbio.1001524)
Supplement: Table S5 — Top gene ontology terms enriched among cluster 3 genes. For Table S5, the 16,766 genes associated with GO terms were compared with the 40 genes in cluster 3 that were associated with GO terms. Listed are the number of genes having that GO term and the number of genes in the cluster having that GO term. (DOCX) [file pbio.1001524.s014.docx]

**Table S5. Top gene ontology terms enriched among Cluster 3 genes**

| GO term | Description | *P* | FDR q | Number in GO | Number in both |
| --- | --- | --- | --- | --- | --- |
| 0019221 | Cytokine-mediated signaling pathway | 2.55 x 10^-18^ | 2.78 x 10^-14^ | 296 | 16 |
| 0071345 | Cellular response to cytokine stimulus | 2.84 x 10^-18^ | 1.54 x 10^-14^ | 371 | 17 |
| 0034097 | Response to cytokine stimulus | 3.92 x 10^-18^ | 1.42 x 10^-14^ | 462 | 18 |
| 0002504 | Antigen processing and presentation of peptide or polysaccharide antigen via MHC class II | 2.14 x 10^-17^ | 5.83 x 10^-14^ | 18 | 8 |
| 0071310 | Cellular response to organic substance | 1.26 x 10^-14^ | 2.74 x 10^-11^ | 1003 | 20 |
| 0002376 | Immune system process | 1.61 x 10^-14^ | 2.91 x 10^-11^ | 1170 | 21 |
| 0034341 | Response to interferon-gamma | 2.13x 10^-14^ | 3.32 x 10^-11^ | 98 | 10 |
| 0060333 | Interferon-gamma-mediated signaling pathway | 3.69 x 10^-14^ | 5.02 x 10^-11^ | 67 | 9 |
| 0006955 | Immune response | 7.12 x 10^-14^ | 8.61 x 10^-11^ | 683 | 17 |
| 0071346 | Cellular response to interferon-gamma | 2.46 x 10^-13^ | 2.68 x 10^-10^ | 82 | 9 |
| 0010033 | Response to organic substance | 6.67 x 10^-13^ | 6.61 x 10^-10^ | 1602 | 22 |
| 0070887 | Cellular response to chemical stimulus | 7.57 x 10^-13^ | 6.87 x 10^-10^ | 1247 | 20 |
| 0019882 | Antigen processing and presentation | 8.15 x 10^-13^ | 6.83 x 10^-10^ | 140 | 10 |
| 0042221 | Response to chemical stimulus | 6.87 x 10^-11^ | 5.35 x 10^-8^ | 2494 | 24 |
| 0050870 | Positive regulation of T cell activation | 1.55 x 10^-10^ | 1.12 x 10^-7^ | 166 | 9 |
| 0002682 | Regulation of immune system process | 2.12 x 10^-10^ | 1.44 x 10^-7^ | 811 | 15 |
| 0031294 | Lymphocyte costimulation | 2.72 x 10^-10^ | 1.74 x 10^-7^ | 70 | 7 |
| 0031295 | T cell costimulation | 2.72 x 10^-10^ | 1.64 x 10^-7^ | 70 | 7 |
| 0051707 | Response to other organism | 3.14 x 10^-10^ | 1.80 x 10^-7^ | 344 | 11 |
| 0050852 | T cell receptor signaling pathway | 9.20 x 10^-10^ | 5.01 x 10^-7^ | 83 | 7 |
| 0002684 | Positive regulation of immune system process | 1.01 x 10^-9^ | 5.25 x 10^-7^ | 495 | 12 |
| 0051251 | Positive regulation of lymphocyte activation | 1.25 x 10^-9^ | 6.18 x 10^-7^ | 210 | 9 |
| 0006952 | Defense response | 1.56 x 10^-9^ | 7.41 x 10^-7^ | 783 | 14 |
| 0050863 | Regulation of T cell activation | 1.60 x 10^-9^ | 7.26 x 10^-7^ | 216 | 9 |
| 0002696 | Positive regulation of leukocyte activation | 2.57 x 10^-9^ | 1.12 x 10^-6^ | 228 | 9 |
| 0050867 | Positive regulation of cell activation | 3.36 x 10^-9^ | 1.41 x 10^-6^ | 235 | 9 |

For Table S5 the 16,766 genes associated with GO terms were compared with the 40 genes in cluster 3 that were associated with GO terms. Listed are the number of genes having that GO term and the number of genes in the cluster having that GO term.
