# Supplementary material for: A Conserved Role for Human Nup98 in Altering Chromatin Structure and Promoting Epigenetic Transcriptional Memory
Source: PLoS Biol. 2013 Mar 26;11(3):e1001524. doi: 10.1371/journal.pbio.1001524 (PMC3608542; doi:10.1371/journal.pbio.1001524)
Supplement: Table S6 — Yeast strains used in this study. (DOCX) [file pbio.1001524.s015.docx]

**Table S6. Yeast strains**

| **Name** | **genotype** | **Fig (s)** | **Reference** |
| --- | --- | --- | --- |
| Spt15-TAP | *MAT***a** *his3∆ leu2∆I met150∆I ura3∆0 SPT15-TAP:His5+* | 1A | Open Biosystems |
| Taf2-TAP | *MAT***a** *his3∆ leu2∆I met150∆I ura3∆0 TAF2-TAP:His5+* | 1B | Open Biosystems |
| Toa2-TAP | *MAT***a** *his3∆ leu2∆I met150∆I ura3∆0 TOA2-TAP:His5+* | 1B | Open Biosystems |
| Sua7-TAP | *MAT***a** *his3∆ leu2∆I met150∆I ura3∆0 SUA7-TAP:His5+* | 1B | Open Biosystems |
| Tfg1-TAP | *MAT***a** *his3∆ leu2∆I met150∆I ura3∆0 TFG1-TAP:His5+* | 1B | Open Biosystems |
| Tfa1-TAP | *MAT***a** *his3∆ leu2∆I met150∆I ura3∆0 TFA1-TAP:His5+* | 1B | Open Biosystems |
| Rad3-TAP | *MAT***a** *his3∆ leu2∆I met150∆I ura3∆0 RAD3-TAP:His5+* | 1B | Open Biosystems |
| Gal11-TAP | *MAT***a** *his3∆ leu2∆I met150∆I ura3∆0 GAL11-TAP:His5+* | 1B | Open Biosystems |
| Kin28-TAP | *MAT***a** *his3∆ leu2∆I met150∆I ura3∆0 KIN28-TAP:His5+* | 1B | Open Biosystems |
| Ctk1-TAP | *MAT***a** *his3∆ leu2∆I met150∆I ura3∆0 CTK1-TAP:His5+* | 1B | Open Biosystems |
| WLY63 | *MAT***a** *ade2-1 can1-100 his3-11,15 leu2-3,112 trp1-1 ura3-1 LEU2:pAFS144 TRP1:pRS304-Sec63-Myc URA3:p6LacO128* | 1C | [1] |
| WLY64 | *MAT***a** *ade2-1 can1-100 his3-11,15 leu2-3,112 trp1-1 ura3-1 LEU2:pAFS144 TRP1:pRS304-Sec63-Myc URA3:p6LacO128::MRS-Kan^r^* | 1C | [1] |
| CRY2 | *MAT*α *ade2-1 can1-100 his3-11,15 leu2-3,112 trp1-1 ura3-1* | 5A,B & F | [2] |
| WLY154 | *MAT***a** *ade2-1 can1-100 his3-11,15 leu2-3,112 trp1-1 ura3-1 ino1-mrsmut* | 5A & B | This study |
| JBY397 | *MAT***α** *ade2-1 can1-100 his3-11,15 leu2-3,112 trp1-1 ura3-1 Sec63-Myc:Kan^r^ LacI-GFP:HIS3 INO1:p6LacO128-INO1* | 5C | [2] |
| WLY155 | *MAT***a** *ade2-1 can1-100 his3-11,15 leu2-3,112 trp1-1 ura3-1 LEU2:pAFS144 TRP1:pRS304-Sec63-Myc INO1:p6LacO128-INO1 set1∆::His5+* | 5C | This study |
| ATY021 | *MAT***a** *ade2-1 can1-100 his3-11,15 leu2-3,112 trp1-1 ura3-1 LEU2:pAFS144 TRP1:pRS304-Sec63-Myc INO1:p6LacO128-INO1 rad6∆::His5+* | 5C | This study |
| VRY61 | *MAT***a** *ade2-1 can1-100 his3-11,15 leu2-3,112 trp1-1 ura3-1 LEU2:pAFS144 TRP1:pRS304-Sec63-Myc* | 5D | [1] |
| WLY149 | *MAT***a** *ade2-1 can1-100 his3-11,15 leu2-3,112 trp1-1 ura3-1 LEU2:pAFS144 TRP1:pRS304-Sec63-Myc set1∆::His5+* | 5D | This study |
| WLY156 | *MAT***a** *ade2-1 can1-100 his3-11,15 leu2-3,112 trp1-1 ura3-1 LEU2:pAFS144 TRP1:pRS304-Sec63-Myc rad6∆::His5+* | 5D | This study |

**Table S6, continued**

| WLY102 | *MAT***a** *ade2-1 can1-100 his3-11,15 leu2-3,112 trp1-1 ura3-1 LEU2:pRS305HA-Htz1 htz1∆::His5+ URA3:p6LacO128::MRS-Kan^r^* | 5E | [1] |
| --- | --- | --- | --- |
| WLY103 | *MAT***a** *ade2-1 can1-100 his3-11,15 leu2-3,112 trp1-1 ura3-1 LEU2:pRS305HA-Htz1 htz1∆::His5+ URA3:p6LacO128::mrsmut-Kan^r^* | 5E | [1] |
| ICY39 | *MAT*α *ade2-1 can1-100 his3-11,15 leu2-3,112 trp1-1 ura3-1 htz1∆::His5+* | 5F | [3] |
| ICY48 | *MAT*α *ade2-1 can1-100 his3-11,15 leu2-3,112 trp1-1 ura3-*1 *htz1∆::His5+ LEU2:pRS305HA-Htz1* | 7A & B | [3] |
| WLY126 | *MAT*α *ade2-1 can1-100 his3-11,15 leu2-3,112 trp1-1 ura3-*1 *htz1∆::His5+ LEU2:pRS305HA-Htz1 nup100∆:: Kan^r^* | 7A & B | [1] |
| WLY166 | *MAT*α *ade2-1 can1-100 his3-11,15 leu2-3,112 trp1-1 ura3-*1 *set3∆::Kan^r^* | 7F | This study |

**References**

1. Light WH, Brand V, Brickner DG, Brickner JH (2010) Interaction of a DNA zip code with the nuclear pore complex promotes H2A.Z incorporation and INO1 transcriptional memory. Mol Cell 40: 112-125.
2. Brickner JH, Walter P (2004) Gene recruitment of the activated INO1 locus to the nuclear membrane. PLoS Biol 2: e342.
3. Brickner DG, Cajigas I, Fondufe-Mittendorf Y, Ahmed S, Lee PC, et al. (2007) H2A.Z-mediated localization of genes at the nuclear periphery confers epigenetic memory of previous transcriptional state. PLoS Biol 5: e81.
