# Supplementary material for: A Conserved Role for Human Nup98 in Altering Chromatin Structure and Promoting Epigenetic Transcriptional Memory
Source: PLoS Biol. 2013 Mar 26;11(3):e1001524. doi: 10.1371/journal.pbio.1001524 (PMC3608542; doi:10.1371/journal.pbio.1001524)
Supplement: Table S7 — Oligonucleotides used in this study. (DOCX) [file pbio.1001524.s016.docx]

**Table S7. Oligonucleotides**

| Name | Sequence |
| --- | --- |
| NP25 For | TCATCCTTCTTTCCCAGAATATTG |
| NP25 Rev | CTCAAATTAACATTGCCGCC |
| GALNB For | CCCCACAAACCTTCAAATTAACG |
| GALNB Rev | CGCTTCGCTGATTAATTACCC |
| INSERT@URA3 For | GCCGCGTTGCTGGCGTTTTTCC |
| INSERT@URA3 Rev | GCCCGCATAGTCAGGAACATCG |
| set1∆ F1 | TTCCTTATTTGTTGAATCTTTATAAGAGGTCTCTGCGTTTAGAGACGGATCCCCGGGTTAATTAA |
| set1∆ R1 | CGATATGTTAAATCAGGAAGCTCCAAACAAATCAATGTATCATCGGAATTCGAGCTCGTTTAAAC |
| set1∆ Check | TCGAAGTACTTCTGCGACACAATGTTGGAC |
| rad6∆ F1 | ATGTCCACACCAGCTAGAAGAAGGTTGATGAGAGATTTTAAACGCGGATCCCCGGGTTAATTAA |
| rad6∆ R1 | CGGCTCGGCATTCATCATTAAGATTCTTTTGATTTTTCGAATTCGAGCTCGTTTAAAC |
| rad6∆ Check | CCCCTAATTGAATTGTTAAAGCGGTC |
| HLA-DRA Pro For | GTTGTCCTGTTTGTTTAAGAAC |
| HLA-Dra Pro rev | TCTTTTGGGAGTCAGTAGAGC |
| HLA-Dra CD For | GAAAGCAGTCATCTTCAGCGTT |
| HLA-DRA CD Rev | AGAGGCATTGGCATGGTGATAAT |
| CIITA Pro For | GTTCCCCCAACAGACTTTCTG |
| CIITA Pro Rev | AGGTGGCCCCAAGCGGTCAG |
| CITIA CD For | CACAGCCACAGCCCTACTTT |
| CIITA CD Rev | CCGACATAGAGTCCCGTGA |
| β Actin Pro For | TTTCGCAAAAGGAGGGGAGAGGGG |
| β Actin Pro Rev | CGCCGCGGCCGCTCGAGCCATAAA |
| β Actin CD For | CTCTTCCAGCCTTCCTTCCT |
| β Actin CD Rev | AGCACTGTGTTGGCGTACAG |
| HLA-DPB1 Pro For | GGGCCAGCAGAATATTTGAGATCACC |
| HLA-DPB1 Pro Rev | GAGTCATTGCTCACTAGGCAGAAAGTTAG |
| HLA-DPB1 CD For | TCCAGCCTAGGGTGAATGTTTCCC |
| HLA-DPB1 CD Rev | TGGTGGACACGACCCCAGCTGTTTCCTCCTG |
| HLA-DQB1 Pro For | GGCACTGGATTCAGAACCTTCACAAA |
| HLA-DQB1 Pro Rev | CTGTGGATGTTTCCATGCGTGGTAGGATTGG |
| HLA-DQB1 CD For | CCCACAGTGACCATCTCCCCATCCAGGAC |
| HLA-DQB1 CD Rev | GGGGTGGACACAACGCCAGCTGTCTCCTCC |
| OAS2 Pro For | CAGTAAACCTTGCTGCAAGGGGCGGGGAAG |
| OAS2 Pro Rev | CCGGGACAGGGAAACAAAACTAACTTAAGC |
| OAS2 CD For | GGCTCCTATGGACGGAAAACAGTC |
| OAS2 CD Rev | CAACCACTTCGTGAACAGACAGAACTTC |
| Nup98 CD For | GGATTATCAGGCTAACAGGAAGGG |
| Nup98 CD Rev | GCCTGAATTAGTGGTGGAGGAGCTG |
| KanB | CTGCAGCGAGGAGCCGTAAT |
| set3F1 | TACAACAGTTTTAGATCGTACTTCACAAAATACGAGAACTGAATCCGGATCCCCGGGTTAATTAA |
| set3R1 | GAATATACTTAAGTTTATATAGGTGTAAGAAGGAAATGTCCATGTGAATTCGAGCTCGTTTAAAC |
| set3Check | GATAAATCCGTTGACTAAGTAGTGGC |
